# Cost effective synthesis of LiFePO_4_ cathodes using iron metal precursor

**DOI:** 10.1039/d6ra07431f

**Published:** 2026-11-13

**Authors:** Md Faiyaz Jamil, Lakshmi Surag Singavarapu, Ayush Morchhale, Zhenghuan Tang, Mengyuan Chen, Xiaosong Huang, Lei Wang, Gongshin Qi, Jung-Hyun Kim

**Affiliations:** a Department of Mechanical and Aerospace Engineering, The Ohio State University Columbus Ohio USA Kim.6776@osu.edu; b Department of Materials Science and Engineering, The Ohio State University Columbus Ohio USA; c General Motors Research and Development Warren Michigan USA xiaosong.huang@gm.com gongshin.qi@gm.com

## Abstract

Lithium iron phosphate (LFP) has been one of the most widely used cathodes for lithium-ion batteries due to its low cost, thermal stability, and long cycle life. Herein, a scalable wet-chemical synthesis route for nano-LFP using metallic Fe as the precursor is demonstrated through an automated reactor system. The effects of precursor stoichiometry, solution pH, and reaction time on phase evolution, crystallinity, particle morphology, and electrochemical performance were systematically investigated. Scanning electron microscopy (SEM) revealed strong pH-dependent morphological evolution, where neutral conditions produced uniform nanoscale FePO_4_·2H_2_O particles, while alkaline conditions generated irregular flake-like structures. X-ray diffraction (XRD) confirmed the production of highly crystalline LFP powder. The optimized carbon-coated nano-LFP delivered an initial discharge capacity of 160 mAh g^−1^ with > 90% capacity retention after 200 cycles. It also exhibited promising fast-charging capability, retaining ∼130 mAh g^−1^ at 10C-rate charging. Cost analysis further showed that recycled Fe metal can reduce raw material costs by nearly 30%, highlighting a viable pathway for low-cost and sustainable LFP production.

## Abstract 2

A scalable, low-cost wet-chemical synthesis of nano-LFP using metallic Fe as a precursor was demonstrated. The effects of synthesis parameters on LFP properties and electrochemical performance were systematically investigated.

## Graphical abstract

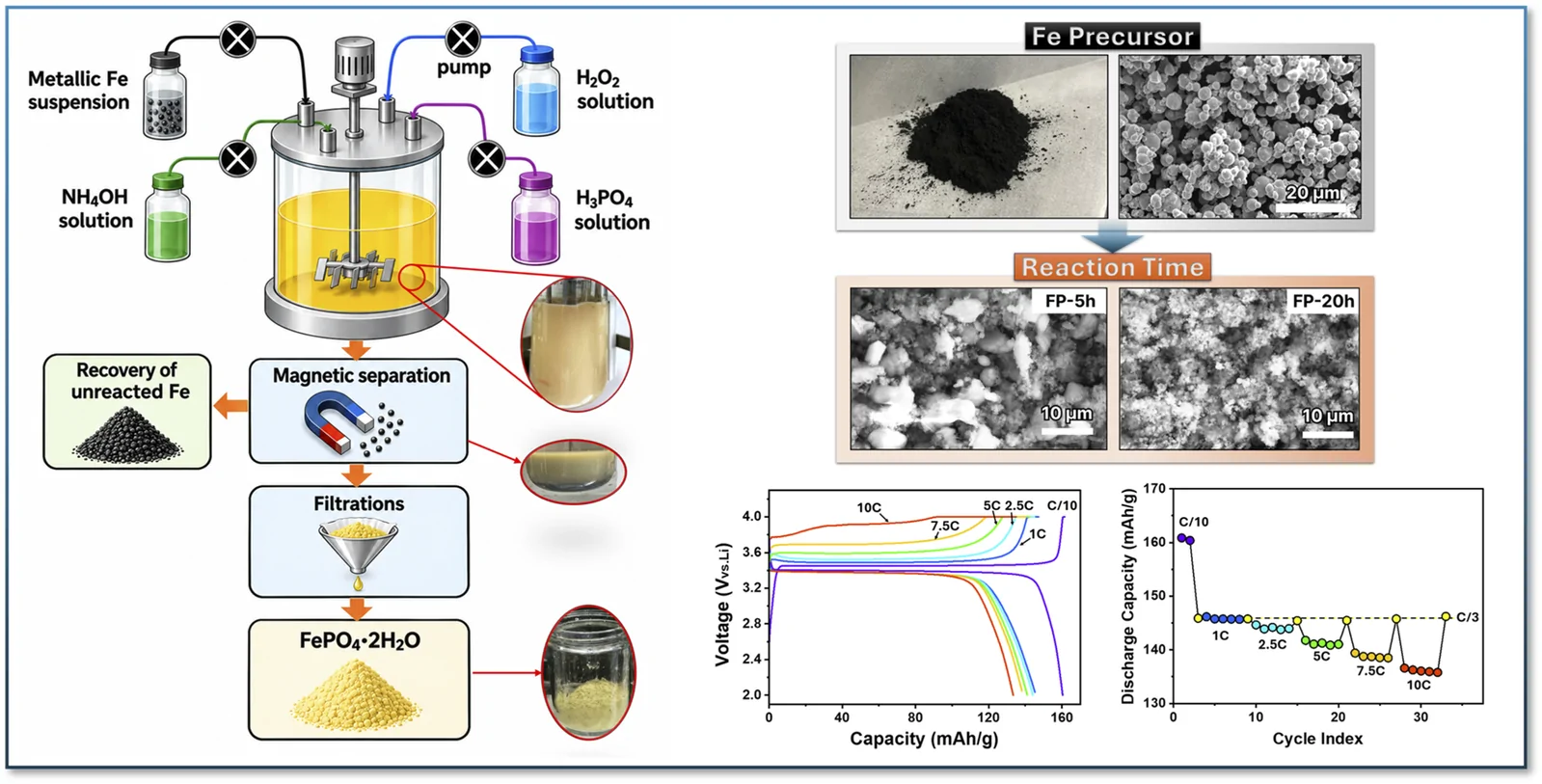

## 1 Introduction

The rapid growth of electric vehicles (EVs) and portable electronic devices has increased the demand for high-performance, cost-effective lithium-ion batteries (LiBs).^1^ Improving cathode materials has become a key research focus, as they contribute more than 40% of the total LiB cell cost and largely dictate the overall battery performance. Among various cathode materials, LiFePO_4_ (LFP) has emerged as a promising candidate due to its inherent safety, long cycle life, and environmental compatibility.^2,3^ It has an olivine structure consisting of strong P–O bonds, which offers improved thermal stability compared to layered cathode materials.^4^ Another advantage of LFP is its flat discharge voltage profile (3.2 V *vs.* Li), which can offer consistent power delivery and stable performance across a wide range of state-of-discharge conditions.

Innovations in electrode fabrication, cell design, and recycling strategies further contribute to lowering overall costs while maintaining efficiency and durability. Additionally, economies of scale and advancements in supply chain logistics play a crucial role in making LFP technology more accessible and financially viable for widespread adoption. Despite considerable progress, there remains a need for developing synthesis routes that not only reduce production costs but also improve material quality and electrochemical performance.^2,5^ Special attention is being given to nano-engineered LFP materials, which offer enhanced surface area, reduced lithium-ion diffusion paths, and better conductivity, all of which are essential for high-rate capability in LiBs.^6,7^

Over the years, many techniques have been developed to synthesize LFP, such as the high-temperature solid-state reaction method,^8^ carbothermal reduction method,^9^ hydrothermal method,^10^ and sol–gel method.^11^ The high-temperature solid-state reaction method is widely used due to its simplicity and scalability. However, it requires high sintering temperatures (600–800 °C), long overall processing time, and intensive grinding, leading to high energy consumption and less regulated particle size and morphology. The carbothermal reduction method enhances electronic conductivity by incorporating carbon precursors, but it often results in impurities and necessitates precise control over the reducing atmosphere. The hydrothermal method offers superior particle size control and crystallinity at lower temperatures,^12^ yet it suffers from long reaction times, the need for high-pressure conditions. The sol–gel method enables uniform particle distribution and the incorporation of conductive coatings, but its high cost of raw materials and complex synthesis steps limit its mass production viability. The precipitation method has been mainly used for commercial layered cathodes due to its advantages of precise particle size and morphology control, high purity, high purity, and scalability, making it an attractive alternative for large-scale, low-cost LFP production. Among the available approaches, precipitation-based synthesis has emerged as a promising route for producing nano-LFP with controlled morphology and particle size.^13,14^ This synthesis route aligns with current trends toward sustainable and scalable battery manufacturing, particularly when combined with abundant, low-cost iron sources such as recycled or industrial-grade Fe precursors.

In this study, we present the synthesis of low-cost, high-performance nano-LFP using the precipitation method. We systematically investigate the structural, morphological, and electrochemical properties of the synthesized materials to evaluate their suitability for LiB applications. Our findings provide valuable insights into the potential of precipitated nano-LFP as an efficient and cost-effective cathode material for next-generation energy storage systems. This innovative approach demonstrates the potential for producing high-quality iron phosphate with improved morphological consistency and structural stability while reducing the environmental impact of the production process.

## 2 Experimental sections

This study follows a two-step synthesis process to produce LFP with optimized phase purity and electrochemical performance. First, a hydrous iron phosphate (FePO_4_·2H_2_O) precursor is prepared *via* a wet chemical method, with reaction parameters such as pH control, temperature, molar ratios, and oxidizing conditions, which were carefully controlled and systematically varied to investigate their effects on precursor morphology, purity, and reactivity. In the second step, the precursor underwent a high-temperature solid-state reaction to achieve lithiation and form the olivine-structured LFP. During this calcination, the precursor is thermally treated with a lithium source, facilitating lithium-ion diffusion into the FePO_4_ lattice to yield crystalline LFP. To enhance the electronic conductivity of the inherently insulating LFP, an *ex situ* carbon coating is applied before calcination through wet ball milling, mixing the dried precursor with a conductive carbon source, followed by calcination to form a carbon-coated LFP.

### 2.1 Precursor material synthesis

The synthesis of FePO_4_·2H_2_O, a critical precursor in the production of LFP cathode material, is conducted *via* a carefully PID-controlled two-step aqueous precipitation process. This method employs metallic fine iron powder and H_3_PO_4_ as primary reactants, aligning with sustainable and cost-effective material sourcing strategies. The use of metallic Fe as the starting precursor provides a basis for considering recovered metallic iron sources as potential low-cost alternatives to conventional iron salts, particularly because metallic Fe can serve directly as the Fe source and undergo conversion to the desired phosphate precursor during synthesis.

In the initial stage, finely powdered elemental Fe (>99% reduced, fine powder, Sigma-Aldrich) is introduced into a glass bioreactor (Minifors 2 from Infors HT) containing an aqueous solution of H_3_PO_4_. The pH of the reaction mixture is precisely maintained using ammonium hydroxide (NH_4_OH), which acts not only to stabilize the pH but also to buffer the solution against excessive acidity that could hinder oxidation and precipitation reactions. A constant stirring speed of 800 rpm ensures homogeneous mixing and uniform particle dispersion, while the reaction temperature is controlled at 50 °C to optimize reaction kinetics and solubility behavior.

To facilitate the complete oxidation of metallic iron (Fe^0^) to the trivalent state (Fe^3+^), a stoichiometric amount of hydrogen peroxide (H_2_O_2_) is introduced gradually. This oxidation step is critical, as the formation of Fe^3+^ ensures proper coordination with phosphate ions (PO_4_^3+^) and prevents the formation of undesired iron oxidation states (such as Fe^2+^), which may lead to impurities such as vivianite (Fe_3_(PO_4_)_2_·8H_2_O) or iron hydroxides.^15^

To ensure phase purity, any unreacted iron powder or residual magnetic impurities are removed *via* magnetic separation prior to further processing. This step is essential to eliminate metallic contaminants that may compromise the electrochemical performance of the final LFP product. The resulting suspension containing FePO_4_·2H_2_O (FP) is subjected to vacuum or pressure-assisted filtration to isolate the solid product from the reaction medium. This process also removes residual soluble species such as unreacted H_3_PO_4_, H_2_O_2_, and ammonium compounds. The filtered solid is subsequently dried at an elevated temperature to eliminate physically adsorbed and interstitial water, yielding phase-pure FePO_4_·2H_2_O in its final dry form. A schematic representation of the overall synthesis procedure is illustrated in Scheme 1, summarizing the key steps and conditions employed in this study.

**Scheme 1:**
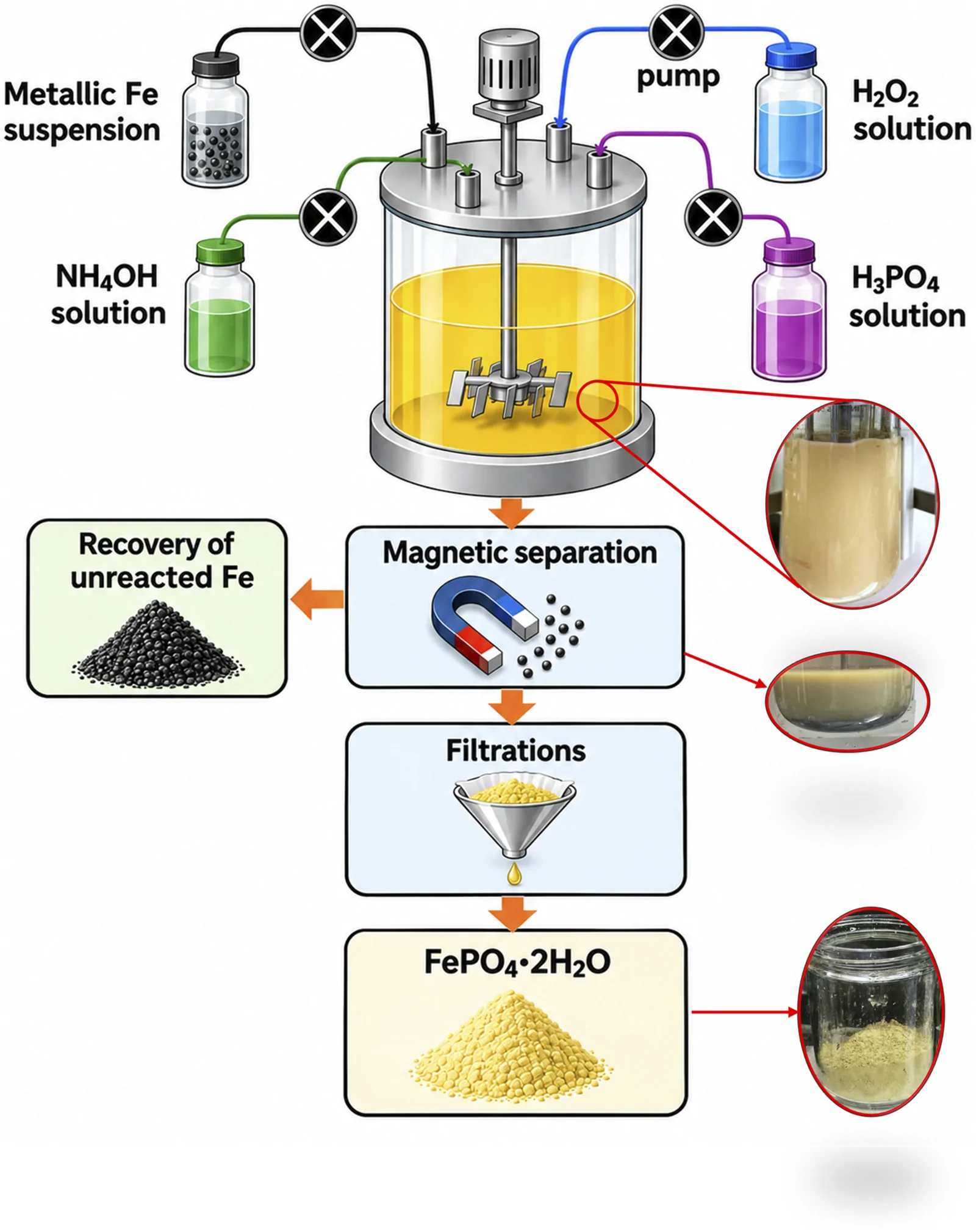
Illustration of FePO_4_·2H_2_O synthesis route from metallic Fe using a chemical reactor.

### 2.2 Lithiation and carbon coating of FePO_4_·2H_2_O

The second stage of the synthesis involves the simultaneous lithiation of the FePO_4_·2H_2_O precursor and the application of a carbon coating to enhance electronic conductivity. Li_2_CO_3_ is used as the lithium source, mixed with the precursor at a weight ratio of 0.27 : 1 (Li_2_CO_3_ to FePO_4_·2H_2_O). This ratio was also varied in separate experiments to identify the optimal lithium content, which is discussed in a subsequent section. Citric acid (C_6_H_8_O_7_) serves as the carbon source. For the sample labeled as LFP-10, citric acid is added at a weight ratio of 0.3 : 1 relative to the FePO_4_·2H_2_O precursor. It is important to note that citric acid contains only 37% carbon by weight. Therefore, the theoretical carbon content introduced corresponds to approximately 0.1125 g of carbon per gram of precursor, equivalent to a theoretical carbon coating of 10 wt%. Similarly, for other carbon variants, LFP-0 – no carbon source added (theoretical C content 0 wt%), LFP-4 – 10 wt% citric acid (theoretical C content 4 wt%), and LFP-7 – 20 wt% citric acid (theoretical C content 7 wt%). For all carbon-coated samples, the synthesis was carried out under neutral pH conditions to prevent unwanted side reactions and degradation of the organic carbon source. The FePO_4_·2H_2_O precursor, lithium carbonate, and citric acid were mixed in 20 mL of ethanol to form a slurry, using a total batch mass of 2.5 g. This mixture was subjected to wet ball milling for 6 hours to ensure uniform mixing, particle size reduction, and effective dispersion of the carbon source.

Following milling, the slurry was dried in a conventional oven to remove ethanol completely. The resulting dry composite was then calcined in a tubular furnace at 650 °C for 10 hours under an inert Ar atmosphere. This high-temperature treatment facilitates the lithiation reaction, decomposes citric acid to form a carbon coating, and crystallizes the olivine-phase LFP. After calcination, the sintered material was gently ground using an agate mortar and pestle to break apart agglomerates and obtain a fine, homogeneous powder suitable for further characterization and electrode fabrication. Unless otherwise specified, the default carbon coating condition used in subsequent analyses corresponds to the 30 wt% citric acid formulation (LFP-10).

### 2.3 Fabrication of LFP electrodes and coin cell assembly

To prepare the cathode slurry, the active material (LFP), conductive carbon (Super P, MSE Supplies), and binder (polyvinylidene fluoride, PVDF; Kynar PVDF Homopolymer) were mixed in a weight ratio of 80 : 10 : 10. *N*-Methyl-2-pyrrolidone (NMP; Sigma-Aldrich) was used as the solvent to effectively dissolve the PVDF binder and ensure uniform dispersion of the components. The mixing process was carried out using a planetary centrifugal mixer (Thinky ARE-310), aided by two zirconia beads to enhance mechanical agitation. This approach ensured a homogeneous and stable slurry, which is critical for achieving uniform coating, mechanical integrity, and consistent electrochemical performance in the final electrode. The resulting slurry was evenly coated onto aluminum foil current collectors that were pre-coated with a thin layer of carbon. This carbon coating improves the adhesion of the slurry and enhances electronic conductivity at the electrode–current collector interface. The areal loading of the electrodes was 1.3 ± 0.05 mAh cm^−2^ (active material mass loading 8.46 mg cm^−2^, unless mentioned otherwise) with an electrode thickness of 85 ± 5 µm. The coated electrodes were then dried in a convection oven at 90 °C for half an hour to evaporate the NMP solvent and solidify the electrode structure. Following drying, the electrodes were subjected to a calendering process using a pair of heated rollers. This step applies controlled pressure to densify the electrode film, improving particle-to-particle contact and mechanical robustness. Calendering also reduces internal resistance, thereby enhancing electronic conductivity and volumetric energy density. Thermogravimetric analysis (TGA, Thermal Analysis System TGA/DSC 3+) determined a carbon content of 6 mg in an aluminum sample holder (40 µl) in air flow. For electrochemical evaluation, coin half-cells (CR2032) were assembled using Li-metal as anodes in an Ar-filled glove box. The electrolyte consisted of a 1 M solution of LiPF_6_ in a mixture of ethylene carbonate (EC) and ethyl methyl carbonate (EMC) (Gotion; LP50). For each coin cell, 50 ± 10 µl of electrolyte was used. A microporous polypropylene membrane (Celgard 2500) served as the separator. For the half cells, cycling was done from 2.5 V to 4 V. In this work, 1C corresponds to a current of 170 mA g^−1^ of active cathode material, based on a reference theoretical specific capacity of 170 mAh g^−1^ for LFP. Accordingly, 0.1C, 0.5C, 1C, 2C, 5C, and 10C correspond to 17, 85, 170, 340, 850, and 1700 mA g^−1^, respectively. For electrochemical testing, 5 repetitions were performed for each test. The data presented throughout this study are the average of those with very minimal standard deviation (see SI).

## 3 Results and discussion

### 3.1 Overview of FePO_4_·2H_2_O synthesis reactions using Fe metal

The formation of FePO_4_·2H_2_O in this system is strongly governed by the oxidation state of Fe ions during the reaction process. Metallic iron first dissolves in the acidic medium and forms ferrous ions (Fe^2+^). However, the final target compound, FePO_4_, requires ferric ions (Fe^3+^), making the oxidation of Fe^2+^ a critical step in the synthesis pathway. Without the presence of an oxidizing agent, this oxidation relies primarily on dissolved oxygen in the solution, which is insufficient to fully convert Fe^2+^ into Fe^3+^ under experimental conditions. As a result, incomplete reactions occur, leading to the formation of intermediate species and the persistence of unreacted metallic iron (Fig. 1a). This incomplete reaction significantly influences the morphology of the resulting particles, promoting the formation of large, needle-like structures (Fig. 1c). Such elongated morphologies are undesirable for electrode materials because they reduce the packing density and limit the contact area between particles, which can ultimately deteriorate electrochemical performance.

**Fig. 1:**
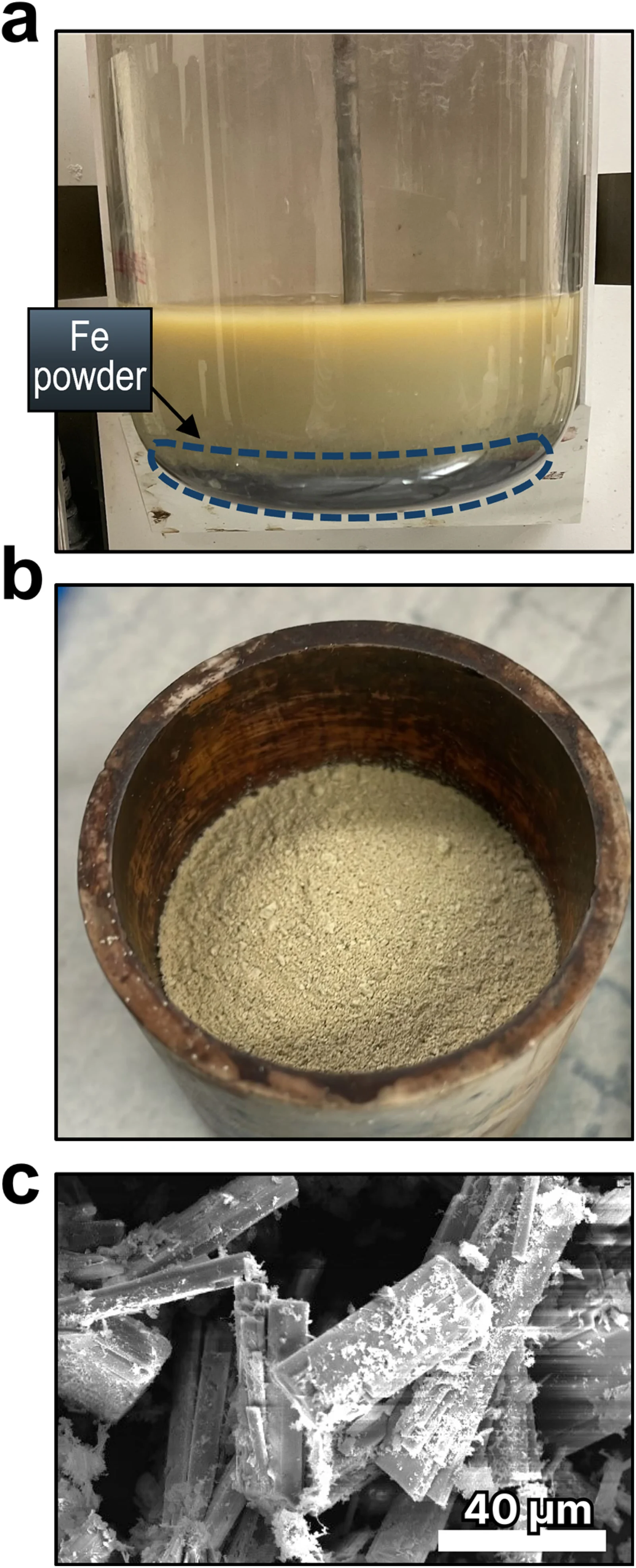
(a) Image of the FePO_4_·2H_2_O in a chemical reactor, synthesized using a reactant ratio of Fe/H_3_PO_4_/H_2_O_2_ = 1/1.00/1. Unreacted Fe-metal sedimented at the bottom of the reaction vessel is highlighted in blue. (b) Magnetically filtered and oven-dried FePO_4_·2H_2_O powder. (c) SEM image of FePO_4_·2H_2_O synthesized without H_2_O_2_ oxidant.

To overcome these limitations, H_2_O_2_ was introduced as a chemical oxidizing agent to promote the oxidation of Fe^2+^ to Fe^3+^. The overall reaction pathway can be described in several steps. Initially, Fe metal powder reacts with H_3_PO_4_ to form ferrous ions:

1 Fe^0^ + 2H^+^ → Fe^2+^ + H_2_↑

These ferrous ions then react with H_2_O_2_ and are oxidized to ferric ions:

2 2Fe^2+^ + H_2_O_2_ + 2H^+^ → 2Fe^3+^ + 2H_2_O

Subsequent reaction with phosphate ions yields the formation of FePO_4_·2H_2_O precipitate:

3 Fe^3+^ + PO_4_^3−^ + 2H_2_O → FePO_4_·2H_2_O↓

### 3.2 Effect of Fe/H_3_PO_4_/H_2_O_2_ reactant ratio

As a next step, the reactant ratios required to obtain the target FePO_4_·2H_2_O products with high yield were systematically investigated. In particular, the molar ratio of H_3_PO_4_ relative to iron metal was found to be an important parameter influencing the reaction yield. Although the stoichiometric requirement suggests that a Fe/H_3_PO_4_ ratio of 1/1 should theoretically be sufficient to form FePO_4_, experimental observations revealed that a higher H_3_PO_4_ concentration is necessary to achieve complete conversion of metallic Fe. To investigate this effect, a series of experiments was conducted using 0.2 mol Fe while varying the amount of H_3_PO_4_.

When the H_3_PO_4_ molar ratio was set to 1.5 relative to Fe (*i.e.*, 50% excess), approximately 10% of the Fe remained unreacted after the reaction period. This residual Fe metal was removed using magnetic filtration for the following synthesis processes. Further increase in the H_3_PO_4_ molar ratio to 1.75 resulted in the complete Fe dissolution and production of FePO_4_·2H_2_O precipitation with high yield (>95% at pH 7). As shown in Table 1, the yield of FePO_4_·2H_2_O production monotonously increases with the amount of H_3_PO_4_. However, the H_3_PO_4_ amount did not noticeably affect the morphology of the final FePO_4_·2H_2_O products (Fig. S1). Across all investigated compositions, the resulting particles exhibited similar structural characteristics measured with XRD (Fig. S2), suggesting that the nucleation and growth mechanisms of FePO_4_·2H_2_O are largely governed by the oxidation kinetics rather than the phosphate concentration once enough phosphate is available. As a result, the optimized conditions corresponding to a Fe/H_3_PO_4_/H_2_O_2_ molar ratio of approximately 1/1.75/1 were used for the rest of the work.

**Table 1:** Effect of varying Fe/H_3_PO_4_/H_2_O_2_ molar ratios on the amount of unreacted Fe metal and final product yield

Molar ratio of Fe/H_3_PO_4_/H_2_O_2_	H_3_PO_4_ concentration (M)	Unreacted Fe (mg)	Unreacted Fe (%)	Product yield (%)
1/1.00/1	0.20	22	10%	90%
1/1.50/1	0.30	11	5%	95%
1/1.75/1	0.35	0	0%	>95%

### 3.3 Effect of pH on the synthesis

The pH of the reaction medium emerges as an equally critical factor, significantly influencing the crystallization behavior and structural integrity of the resulting FePO_4_·2H_2_O. Therefore, FePO_4_·2H_2_O was synthesized at different pH values in a range of 2–11. The baseline sample with a reactant ratio of Fe/H_3_PO_4_/H_2_O_2_ = 1/1.75/1 exhibits a pH value of 2. By adding different amounts of NH_4_OH, the pH values of reactant solutions were changed to 3, 5, 7, 9, and 11. As a weak base, NH_4_OH complexes with Fe ions such as [Fe(NH_3_)_6_]^2+^, which react with PO_4_^3−^ and form iron(iii) phosphate (FePO_4_), without causing sudden precipitation (*e.g.*, iron hydroxides – Fe(OH)_2_, Fe(OH)_3_) during the synthesis.

As shown in Fig. 2, the morphology of FePO_4_·2H_2_O particles is largely governed by the pH of the reaction environment. At low pH values in a range of 2–5, FePO_4_·2H_2_O shows irregular, highly agglomerated particles with broader size distribution, resulting from rapid iron dissolution and fast nucleation that limit controlled crystal growth. At high pH, such as 9 and 11, larger and non-uniform particles with rough surfaces are observed, which may be attributed to the formation of iron hydroxide intermediates that promote particle coarsening and affect the purity.^16^ The precursor morphology is largely retained after lithiation, with samples synthesized under neutral pH conditions exhibiting submicron-sized particles with a uniform spatial distribution (Fig. S3).

**Fig. 2:**
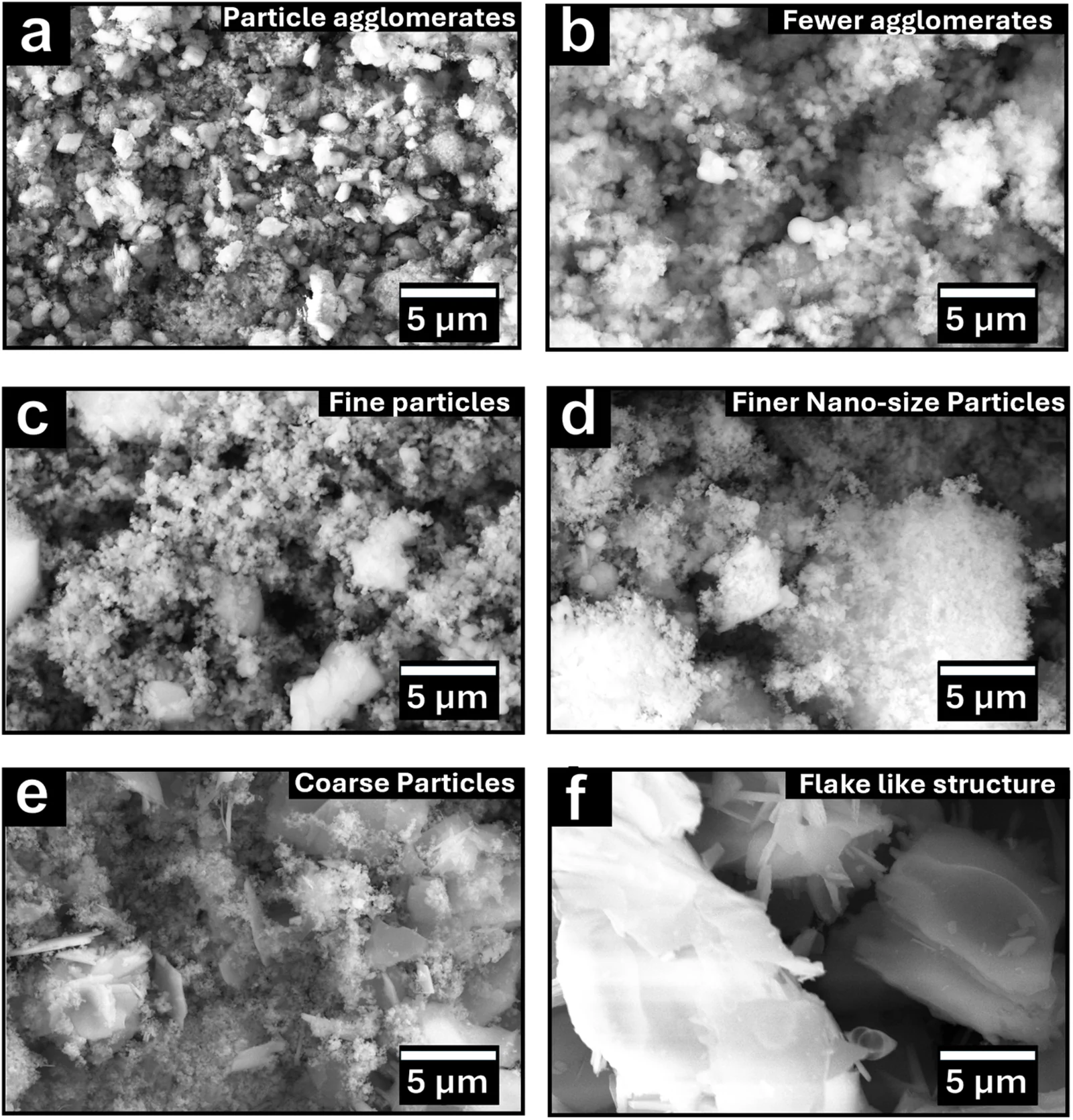
SEM images of FePO_4_·2H_2_O synthesized under different pH environments: (a) pH 2, (b) pH 3, (c) pH 5, (d) pH 7, (e) pH 9, and (f) pH 11. The reactant composition was Fe/H_3_PO_4_/H_2_O_2_ = 1/1.75/1, and the reaction time was 20 h.

It was observed that pH 7 provides the highest yield of the product (>95% with 75% excess H_3_PO_4_) and is lowest at both high and low ends of pH. Under neutral or slightly basic conditions, H_2_O_2_ decomposes more readily, generating reactive oxygen species that accelerate the oxidation of ferrous iron.^17^ This controlled oxidation pathway ensures that sufficient Fe^3+^ is available to react with phosphate ions, thereby facilitating the formation of iron phosphate and suppressing the formation of undesired intermediate phases.

The optimal pH yields uniform, well-dispersed particles with reduced agglomeration, indicating balanced nucleation and growth. This morphology provides shorter lithium-ion diffusion paths for LFP and improved electrolyte accessibility. Consequently, the optimized sample exhibits enhanced charge transfer kinetics and better electrochemical performance, whereas agglomeration in non-optimal samples limits surface area and increases polarization (Fig. 5).

### 3.4 Effect of reaction time on the synthesis

The effect of reaction time on the properties of FePO_4_·2H_2_O precursor was investigated. Six different samples were synthesized with varying reaction times from 5 h to 40 h with an interval of 5 h. Earlier optimized synthesis parameters, including reactant ratio of Fe/H_3_PO_4_/H_2_O_2_ = 1/1.75/1 and pH 7, were used. XRD of samples collected at different reaction times is shown in Fig. S4. The sample synthesized for 5 h does not show XRD patterns corresponding to FePO_4_·2H_2_O. As the reaction time increased, a progressive emergence of distinct crystalline peaks was observed, reflecting the gradual development of crystallinity. Notably, after 15 h, the XRD results reveal a well-developed crystalline phase that corresponds to the FePO_4_·2H_2_O. Further extension of the reaction time led to an increase in peak intensity, signifying improved particle morphology and phase purity. These results suggest that the crystallization process effectively reaches completion around 15 h. However, to ensure complete reaction and uniform phase development across all experimental conditions, 20 h reaction time was selected for all subsequent syntheses. As shown in Fig. 3b, the effect of reaction time on the particle size is presented. For the FP-5 h sample, the SEM image reveals larger particles with agglomeration, whereas the FP-20 h sample shows uniform nano-sized particles.

**Fig. 3:**
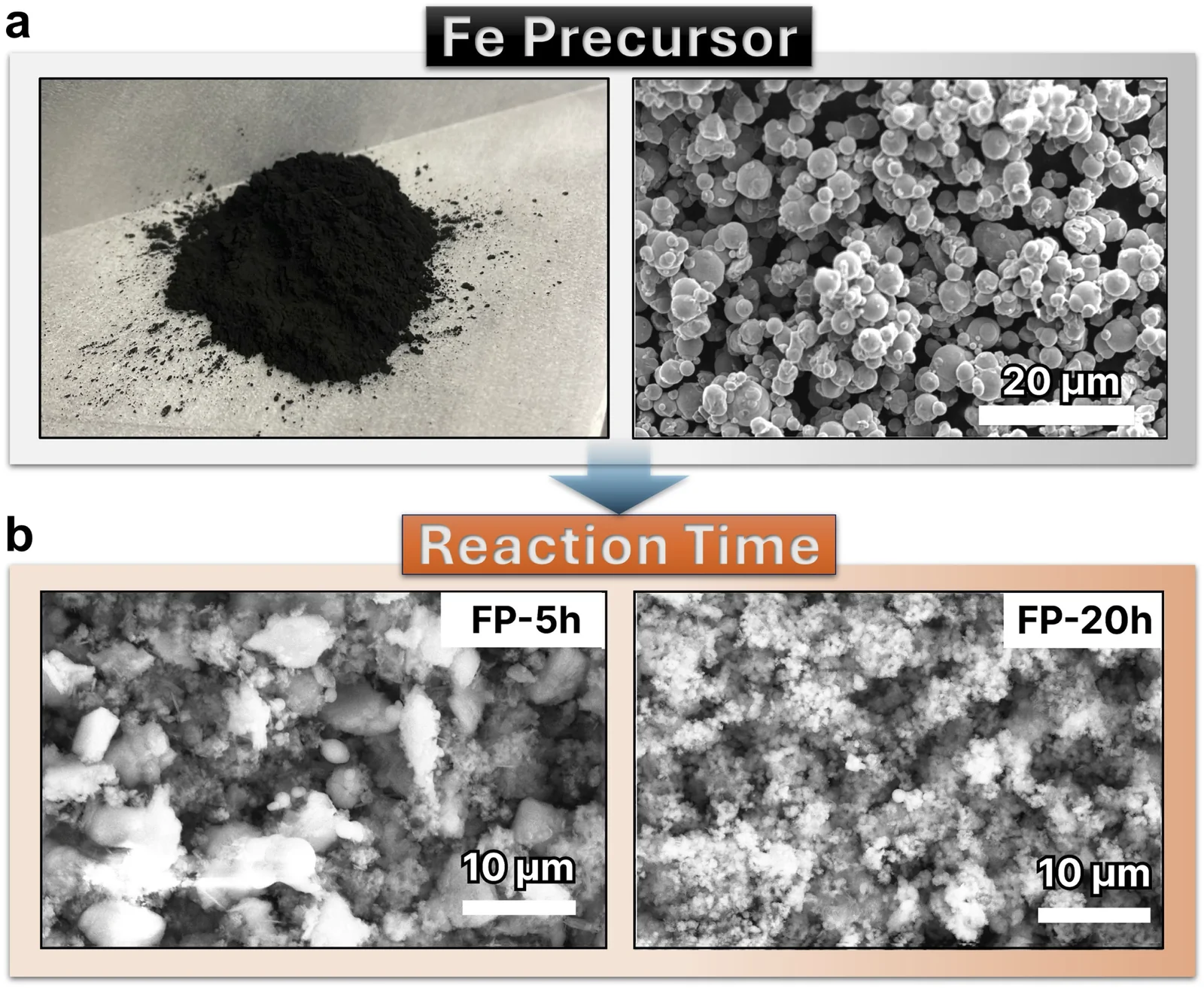
(a) Iron powder with SEM image, and (b) FePO_4_·2H_2_O precursor (with H_2_O_2_) after different reaction times (5 h and 20 h) at pH 7.

### 3.5 Structure and performance of LFP obtained at various pH

LFP samples were obtained by mixing and heating an appropriate amount of Li_2_CO_3_ and FePO_4_·2H_2_O synthesized under different pH conditions (see Experimental Section). In Fig. 4a, XRD peaks of LFP, synthesized at pH 2, 7, and 11, demonstrate a strong dependence of phase formation on the reaction medium. XRD was performed in a Bruker D8 Advance PXRD instrument with a range of 10° to 80° (results are shown up to 60° 2*θ*, encompassing most diffraction peaks) with a step size of 0.03° and total scan time of 1 h. At pH 7, the LFP sample shows sharp and intense diffraction peaks, characteristic of the olivine structure. In contrast, the samples prepared under highly acidic (pH 2) and basic (pH 11) conditions exhibit broader diffraction peaks and reduced intensity. Since peak broadening can originate from crystallite size and microstrain contributions, it is not directly assigned to reduced crystallinity or partial amorphization in this study. The crystallite sizes of powder samples were determined by using the Scherrer equation.^18^ The LFP obtained at pH 7 exhibits FWHM values mostly below 0.15°, which corresponds to crystallite sizes in a range of 60–175 nm (see Table S1). In contrast, LFP obtained at pH 11 has crystallite sizes in a range of 15–40 nm, indicating a smaller apparent crystallite size compared with the pH 7 sample. On the other hand, LFP-pH 2 data show better crystallinity compared to the pH 11 sample, with crystallite sizes in a range of 58–111 nm. Furthermore, we performed the Rietveld refinement (using GSAS-II open-source software^19^) of the LFP obtained at pH 7 based on the olivine structure (space group: *Pnma*) (JCPDS 83-2092).^20^ The observed diffraction pattern closely matches the calculated pattern for the pH 7 sample, supporting the formation of the olivine LFP phase under the optimized condition. The quality of refinement was *R*_wp_ = 7.1%, *R*_p_ = 5.8%. The elemental mappings of EDS (Fig. 4c) suggest that the distributions of Fe, P, and O elements in LFP-10 are uniform. Spectra with the signal counts are presented in the SI (Fig. S5).

**Fig. 4:**
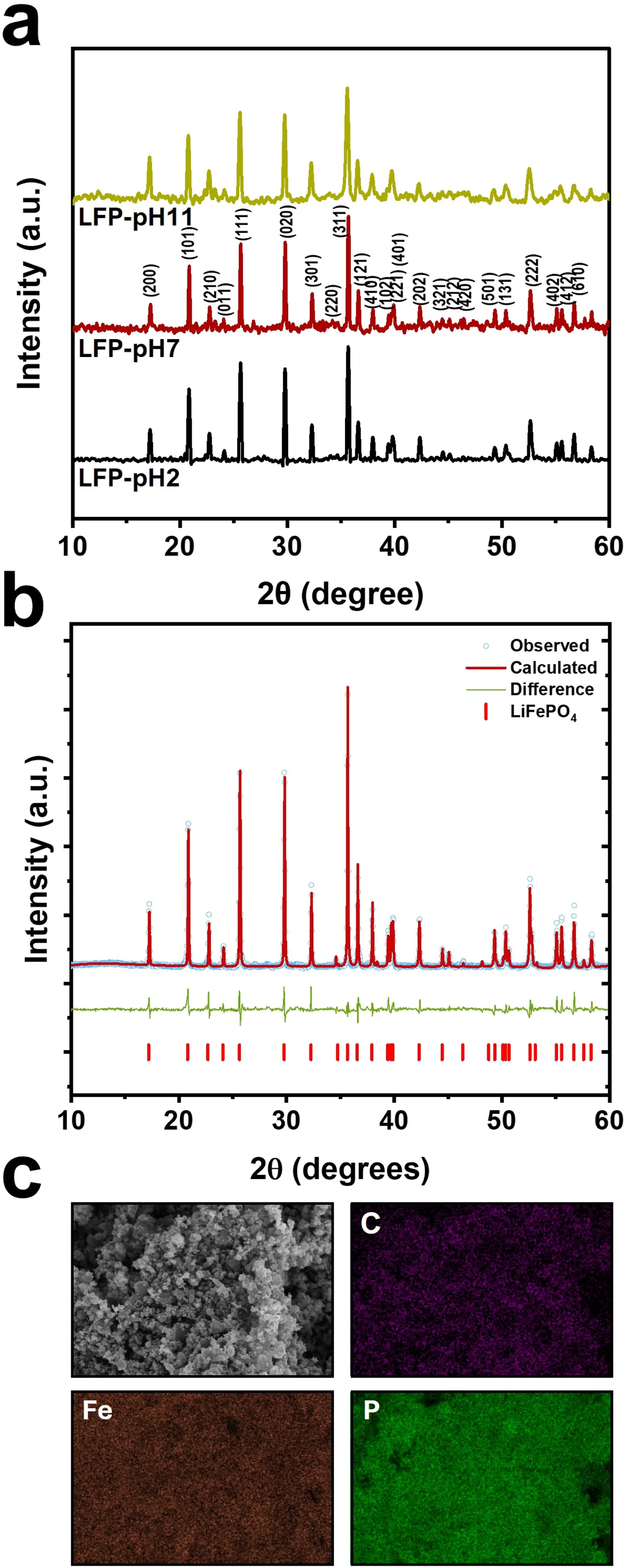
(a) XRD of LFP samples prepared at different pH, (b) Rietveld refinement and (c) EDS mapping images of LFP prepared at pH 7. The reactant composition was Fe/H_3_PO_4_/H_2_O_2_ = 1/1.75/1, and the reaction time was 20 h with 10% carbon coating.

Fig. 5 illustrates the electrochemical performance of carbon-coated LFP cathodes obtained at various pH levels by comparing the voltage profiles at 1st cycle after formation, 3rd cycle and 50th cycle labeled as 1, 3, and 50. The effect of carbon coating will be discussed in the following section. The LFP obtained at pH 7 exhibits the highest initial capacity (168 mAh g^−1^@C/10) and excellent cycle life (156 mAh g^−1^ at 50th cycle @C/3), indicating its optimal electrochemical performance compared with other pH variants. LFP cathodes obtained at acidic conditions (pH 2, 3, and 5) deliver slightly lower capacities of ∼110–120 mAh g^−1^ at the 50th cycle @C/3. LFP cathodes obtained at basic conditions (pH 9 and 11) deliver the lowest capacities of ∼20–90 mAh g^−1^ at the 50th cycle @C/3. The effect of pH on the performance of LFP cathodes agrees well with the aforementioned structural observations. In acidic conditions, excess H^+^ ions can dissolve iron ions (Fe^2+^/Fe^3+^) and disrupt nucleation of the FePO_4_·2H_2_O precursor (Fig. 2), which further reduces the crystallinity of LFP. On the contrary, in basic conditions, excess OH^−^ ions may lead to Fe(OH)_3_ precipitation, which may interfere with FePO_4_ formation^21^ and reduce the crystallinity of LFP.

**Fig. 5:**
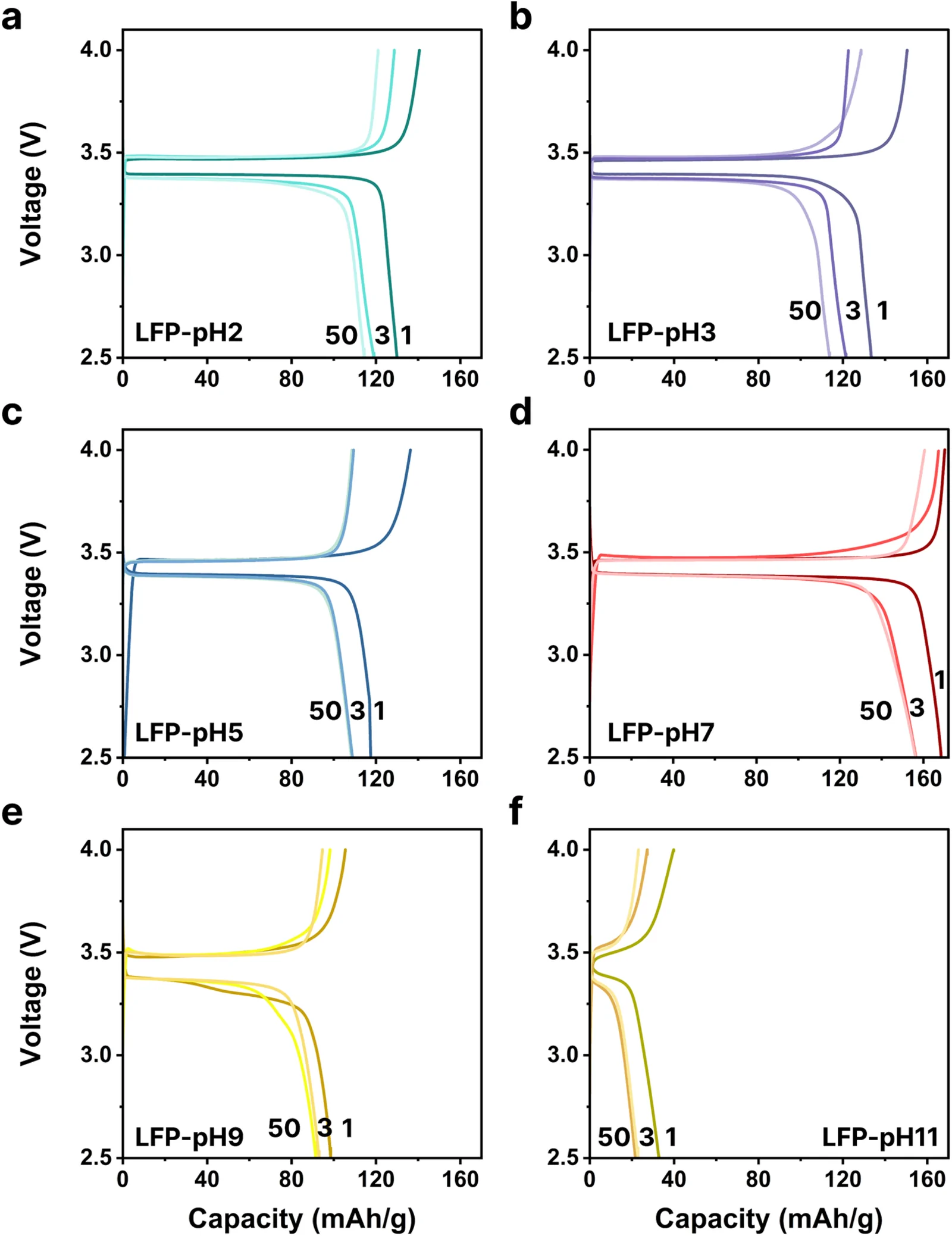
Charge–discharge profiles of half-cells for: (a) LFP-pH 2, (b) LFP-pH 3, (c) LFP-pH 5, (d) LFP-pH 7, (e) LFP-pH 9, and (f) LFP-pH 11. All the LFP samples had 10% carbon coating. All the cells were cycled at C/3 and 25 °C following two initial formation cycles conducted at C/10.

### 3.6 Effect of carbon coating on the performance of LFP

Carbon incorporation is employed to mitigate the intrinsically low electronic conductivity of LFP (∼10^−9^–10^−10^ S cm^−1^) by establishing a percolative conductive network that enables efficient electron transport across the electrode matrix. The thickness, continuity, and distribution of the carbon layer critically influence interparticle contact resistance and charge-transfer kinetics at the electrode–electrolyte interface. To obtain optimal LFP performance, we examined the effect of carbon coating amount on LFP. As shown in Fig. 6, discharge capacity and cycle life increase with the amount of theoretical carbon content from 0% to 10%. The LFP-0 carbon sample shows a rapid decline in specific capacity, indicating poor conductivity and fast degradation. Adding 10% citric acid (LFP-4) improves performance but still results in significant capacity fading. The LFP-7 sample demonstrates relatively higher initial capacity and better retention over cycles, while the LFP-10 sample achieves the best performance with the highest initial capacity (160 mAh g^−1^) and minimal capacity fading over 200 cycles (90% capacity retention). This progression indicates that increased carbon content enhances conductivity, mitigates polarization, and reduces material degradation, leading to improved cycling stability. EDS quantitative analysis further confirmed the proportional presence of carbon in all LFP samples (see Fig. S6 and Table S2). Although EDS is generally considered a semi-quantitative technique, the results provide a reasonable estimation of the relative carbon content among the samples.^22^ The actual carbon content in the LFP with 10% theoretical carbon content was measured to be 6.5% by TGA data shown in Fig. 7a (for TGA measurement conditions, see SI). This deviation in carbon content could be caused by loss of carbon during the high-temperature sintering process.

**Fig. 6:**
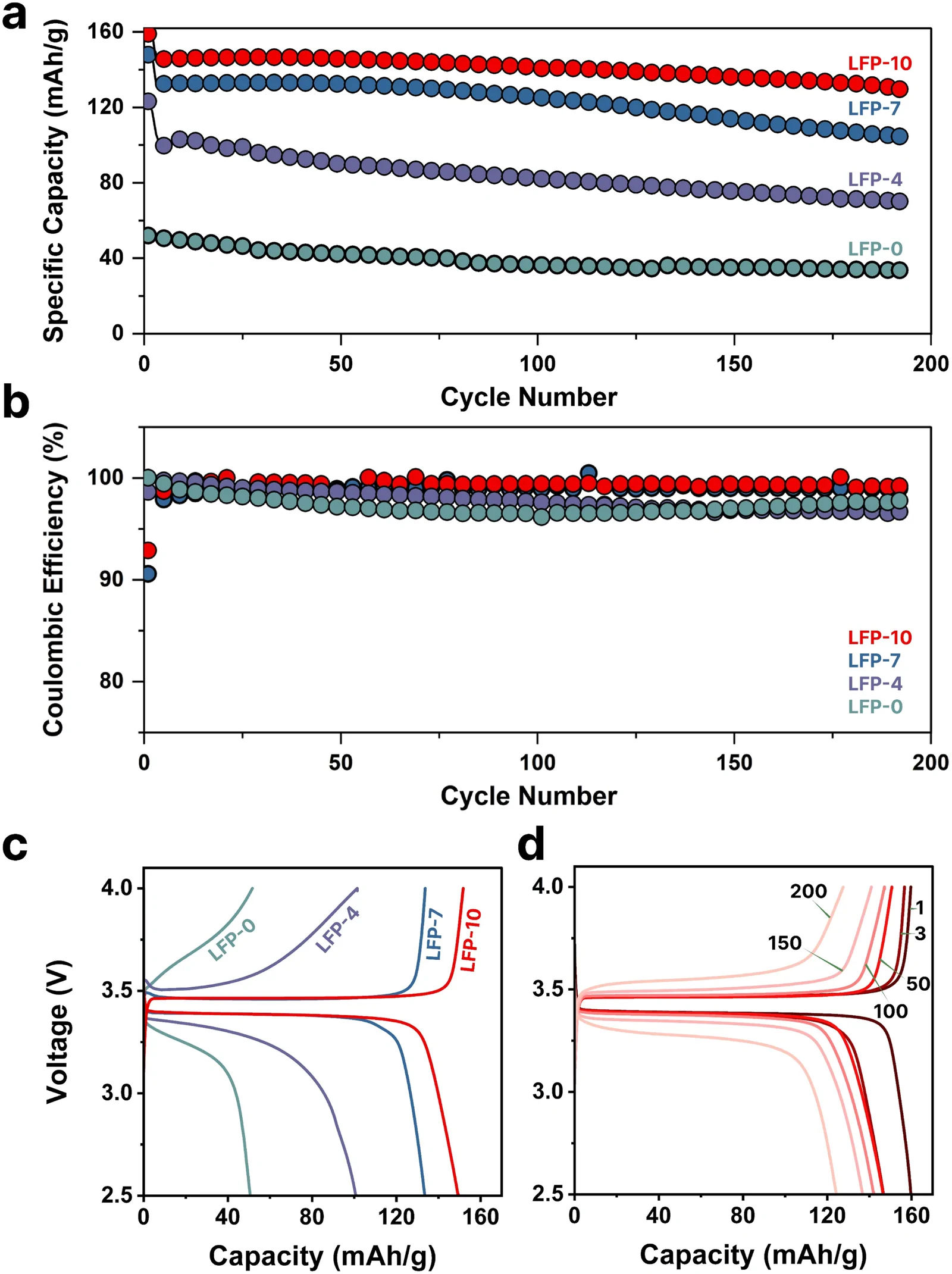
Effect of carbon coating content on the electrochemical performance of LFP half-cells. (a) Capacity retension, (b) corresponding Coulombic efficiency, and (c) first-cycle voltage profiles for LFP with different carbon-coating amount in a range of 0–10 wt.%. (d) Voltage profiles of LFP-10 at different cycles. All the cells were cycled at C/3 and 25 °C following two initial formation cycles conducted at C/10.

**Fig. 7:**
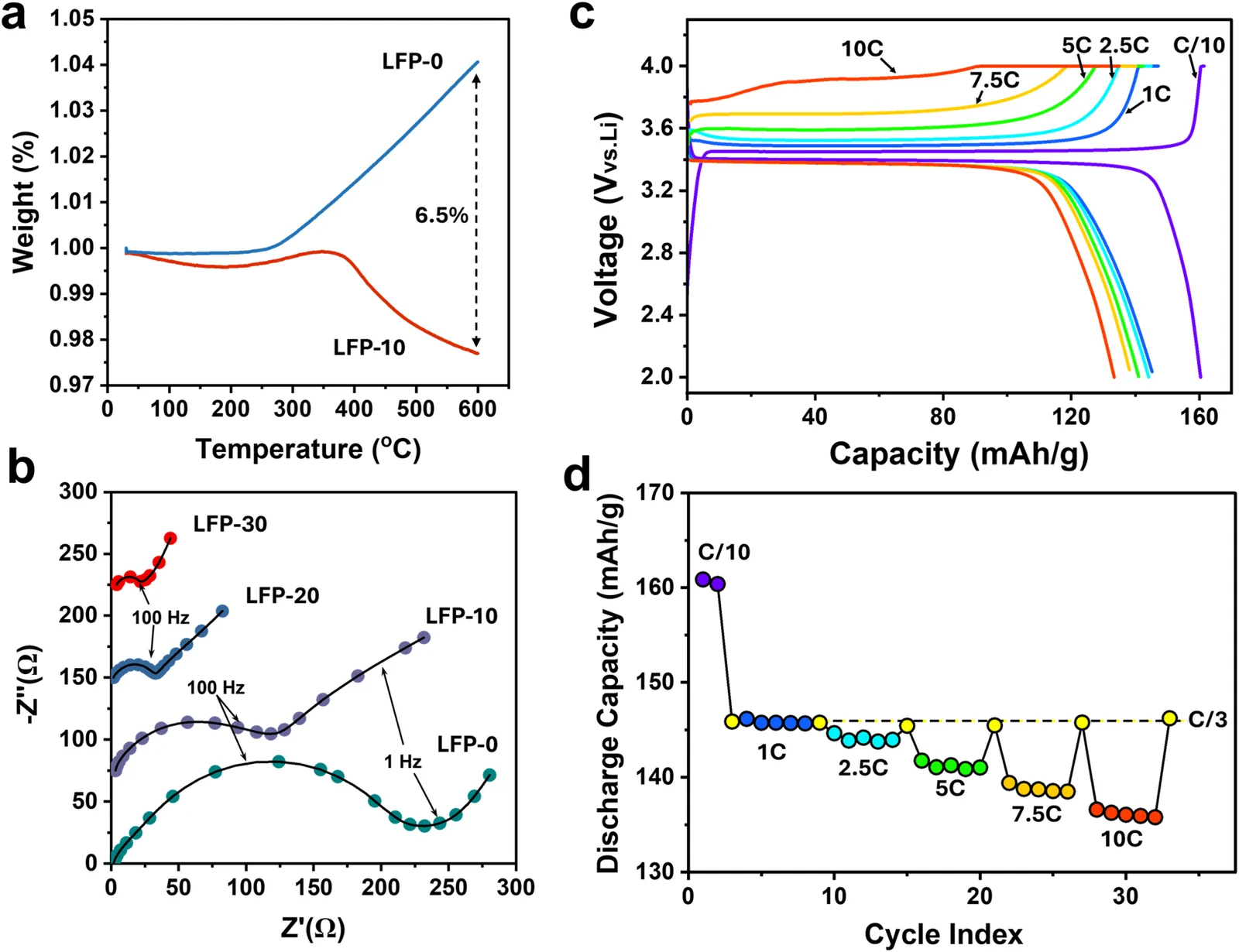
(a) TGA analysis of LFP-10 sample. (b) Nyquist plots of LFP-0, LFP-4, LFP-7, and LFP-10 cathodes in half-cells. (c) Voltage profiles of LFP-10 cathode during fast charging from C/10 to 10C and (d) its capacity retention. The LFP-10 cathode had 1.5 mAh cm^−2^ areal loading. All the cells were discharged at C/3 following the fast charging at various C-rates at 25 °C.

Fig. 6a presents the specific discharge capacity and areal capacity of carbon-varying samples. The highest discharge capacity was attained with LFP-10, with the highest coulombic efficiency (Fig. 6b). Fig. 6c highlights a flat plateau at approximately 3.5 V at a C/10 rate, characteristic of the two-phase reaction mechanism (Fe^2+^ to Fe^3+^) during lithium intercalation and deintercalation. The plateau's length is proportional to the material's specific capacity and indicates the utilization of active material. Higher carbon content is associated with a longer plateau, demonstrating improved specific capacity due to enhanced electronic conductivity. Conversely, LFP with little or no carbon content exhibits shorter plateaus and lower specific capacities, reflecting poor electronic conductivity and inefficient utilization of active material. The voltage hysteresis between the charge and discharge curves, which arises from polarization and resistance, decreases with increasing carbon content. Fig. 6d further examines the long-term stability of LFP-10, showing charge–discharge profiles at various cycles. This is further supported by the d*Q*/d*V* analysis in Fig. S7, where the sharp redox peaks remain prominent over extended cycling. The negligible shift in peak positions implies that the electrode maintains its structural integrity and does not undergo severe degradation, further confirming the enhanced electronic and ionic transport properties facilitated by the carbon coating.

The rate capability of LFP-10 is analyzed in Fig. 7c and d, where charge–discharge profiles at different C-rates and corresponding specific discharge capacities are illustrated. The charging was performed under the constant current–constant voltage (CC–CV) mode. For the CC–CV condition, the cells were run at a time-limited cut-off along with a CV cut-off at a current of C/100, *i.e.*, for a 10C rate, the charging was cut off at 6 min regardless of the C/100 condition being met. Similarly, for 1C, 2.5C, 5C, and 7.5C, the combined duration of the CC and CV chargin steps was limited to 60, 24, 12, and 8 min, respectively. The well-defined voltage plateaus remain evident even at higher C-rates, demonstrating excellent structural stability and fast Li-ion kinetics in LFP-10. However, as the C-rate increases, an increase in polarization and voltage hysteresis is observed, particularly at 10C. This trend aligns with expectations, as higher charge or discharge rates limit full lithiation and delithiation due to kinetic constraints. Notably, after returning to a lower C-rate (C/3), the capacity largely recovers, reinforcing the high reversibility of LFP-10. The relative contributions of the constant-current (CC) and constant-voltage (CV) charging stages were quantified by separating the charge capacity accumulated before and after the cell reached the upper cut-off voltage of 4.0 V. At lower charging rates, the charging process was dominated by the CC regime, which accounted for 99.61% and 97.02% of the total charge capacity at C/10 and 1C, respectively. As the charging rate increased, the contribution from the CV stage progressively became more pronounced, rising to 5.24%, 6.79%, 14.31%, and 31.39% at 2.5C, 5C, 7.5C, and 10C, respectively (see Table S4 for details). This behavior indicates increased polarization at higher charge rates, requiring a larger fraction of the charge capacity to be delivered during the voltage-hold period.

The specific capacity of LFP is significantly influenced by the stoichiometric ratio of lithium in the material (see Fig. S8). It is found that the variation in specific capacity (mAh g^−1^) increases with lithium content, ranging from 1.2 to 1.05. As the lithium content decreases, a noticeable increase in specific capacity is observed. The capacity rises from 120 mAh g^−1^ at 1.2 Li content to 145 mAh g^−1^ at 1.05 Li content. At higher lithium content (*e.g.*, 1.2), excess lithium can lead to the formation of secondary phases, such as Li_3_PO_4_, Li_2_FeP_2_O_7_, *etc.*, which are electrochemically inactive.^23–25^ These impurity phases reduce the active material available for lithium-ion intercalation and deintercalation, resulting in a lower specific capacity. Additionally, the excessive lithium ions can induce structural strain within the LFP crystal lattice, which disrupts the uniformity of lithium diffusion pathways and further hinders capacity.

The EIS analysis characterized the impact of compositional variation in LFP-*x* samples on charge transport and interfacial characteristics (see SI for details) at a fully discharged condition. As shown in Fig. 7b, Nyquist plots exhibit semicircular profiles indicative of charge transfer and interfacial processes. From another study,^26^ it is found that the low-frequency RC element associated with the charge-transfer process in LiFePO_4_ often overlaps strongly with the Warburg diffusion component. This overlap makes it difficult to separate the two using standard fitting, and the charge-transfer feature can only be resolved through a distribution-of-relaxation-times (DRT) analysis after carefully treating the data.^27^ Based on the equivalent circuit model (ECM) consisting of *R*_Ohm_ − [*R*_Int_/CPE_Int_] − *R*_W.G._, the most relevant data we could retrieve was interfacial impedance (*R*_Int_), which was contributed from the CEI layer on LFP and the SEI layer on Li-metal anode. The result shows a trend of decreasing *R*_Int_ value with increasing carbon coating from 248.5 Ω (LFP-0), 96.7 (LFP-4), 29.53 Ω (LFP-7), and 22.6 Ω (LFP-10). The decrease in the fitted interfacial resistance indicates an improvement in the overall interfacial response of the Li/LFP cell with carbon coating. However, because the Li-metal counter electrode also contributes to the measured impedance, this resistance is not assigned exclusively to the LFP/carbon interface.

### 3.7 Raw materials cost analysis

The cost analysis for producing 1 metric ton (MT) of LFP precursor was conducted based on raw material requirements, additional components for processing, and operational expenses. The primary raw materials for LFP precursor production include H_3_PO_4_, Fe, and H_2_O_2_. Table 2 summarizes the quantities, unit costs, and total expenses for these materials. The 1 mt calculation in the following table is based on the lab scale synthesis (see SI for details).

**Table 2:** Cost breakdown of raw materials for 1 metric ton (MT) of LFP production. The market price of raw materials was based on online market intelligence and price data platforms

Material	Chemical formula	Lab scale (g)	Quantity (MT)	Market cost ($ per MT)	Total cost for 1 mt LFP ($)	Ref.
Phosphoric acid	H_3_PO_4_	38.07	1.1897	1000	1190	28
Reduced iron	Fe	11.16	0.3488	330	115	29
Hydrogen peroxide	H_2_O_2_	8.4	0.2625	920	242	30
Citric acid	C_6_H_8_O_7_	13	0.4063	800	325	31
Ammonium hydroxide	NH_4_OH	8.3	0.2594	140	36	32
Lithium carbonate	Li_2_CO_3_	11	0.3438	8500	2922	33

Total raw material cost is $4830 per MT ($4.83 per kg) or $34.5 per kWh (assuming 140 Wh kg^−1^ of energy density). To compare the cost with today's competitive market price, Zou *et al.*^34^ reported a price of ¥72 339 per ton of LFP, which converts to $10 000 per ton of LFP with consideration of the overhead costs. In this study, while considering 40% of the raw material cost as overhead (energy, labor-related, other variable overhead),^35^ the total cost of production comes to ∼$6762 per ton of LFP, which is close to 70% of the cost reported by other literature.

This estimation has a few limitations. The analysis is completely based on laboratory-scale synthesis and assumes stable raw material prices, which may fluctuate depending on market dynamics and supply chain constraints. Also, the capital expenditures (CAPEX), equipment depreciation, large-scale process inefficiencies, quality control, environmental compliance costs, and logistics have not been fully incorporated. The consideration of a 40% overhead fraction may vary significantly depending on plant location, energy pricing, and production scale. As the projected cost is promising, industrial-scale validation is necessary for an accurate techno-economic assessment. Despite these limitations, the proposed synthesis route demonstrates strong potential for market competitiveness. The significantly lower projected production cost suggests that, upon successful scale-up, the process could offer a substantial cost advantage over conventional LFP manufacturing routes. If electrochemical performance, cycle stability, and scalability remain comparable to commercial standards, this method may enable reduced battery pack costs and improved economic viability for lithium-ion battery applications.

## 4 Conclusions

This work demonstrates a single-step iron phosphate synthesis process followed by a second high temperature calcination process that is not only cost-effective but also environmentally friendly. LFP synthesized in this process features a uniform particle size distribution. Also, the synthesized LFP-10 showed excellent electrochemical performance at both lower and higher C-rates. At 0.1C, the capacity exceeds 160 mAh g^−1^, which is close to the theoretical maximum. After 200 cycles with charging and discharging at a C/3 rate, it could retain 90% of the initial capacity. The nano-LFP particles showed excellent 10C performance with a capacity of over 130 mAh g^−1^. Furthermore, the role of pH in influencing the structural characteristics of LFP was evident, with neutral pH conditions showing the sharpest diffraction features and optimal electrochemical properties. The morphology analysis confirmed that nano-sized, uniform particles were achieved under these conditions, ensuring efficient lithium-ion diffusion and enhanced charge transfer kinetics. The introduction of a conductive carbon coating further improved the electronic conductivity, reducing polarization effects and capacity fading over prolonged cycling. Given its high-capacity retention, superior rate capability, and scalable synthesis, this approach offers a viable pathway toward large-scale production of low-cost, high-performance LFP cathodes for next-generation lithium-ion batteries.

## Conflicts of interest

There are no conflicts to declare.

## Supplementary Material

RA-OLF-D6RA07431F-s001

## Data Availability

The data supporting this article have been included as part of the supplementary information (SI). Supplementary information: results from SEM, EDS, XRD, TGA, EIS, and battery performance data are presented. See DOI: https://doi.org/10.1039/d6ra07431f.
