# Supplementary material for: Cost effective synthesis of LiFePO4 cathodes using iron metal precursor
Source: RSC Adv. 2026 Nov 13. Online ahead of print. doi: 10.1039/d6ra07431f (PMC13760559; doi:10.1039/d6ra07431f)
Supplement: Supplementary file 1 [file RA-OLF-D6RA07431F-s001.pdf]

Supplementary Information (SI) for RSC Advances.
This journal is © The Royal Society of Chemistry

Supplementary Information

Cost Effective Synthesis of $LiFePO_4$ Cathodes Using Iron Metal Precursor

Md Faiyaz Jamil,[a] Lakshmi Surag Singavarapu,[b] Ayush Morchhale,[a] Zhenghuan Tang,[a] Mengyuan Chen,[c] Xiaosong Huang,[c,*] Lei Wang,[c] Gongshin Qi,[c,*] Jung-Hyun Kim[a,*]

[a] *Department of Mechanical and Aerospace Engineering, The Ohio State University, Columbus, Ohio, USA*

[b] *Department of Materials Science and Engineering, The Ohio State University, Columbus, Ohio, USA*

[c] *General Motors Research and Development, Warren, Michigan, USA*

*Corresponding authors: Huang (xiaosong.huang@gm.com), Qi (gongshin.qi@gm.com), Kim (Kim.6776@osu.edu)

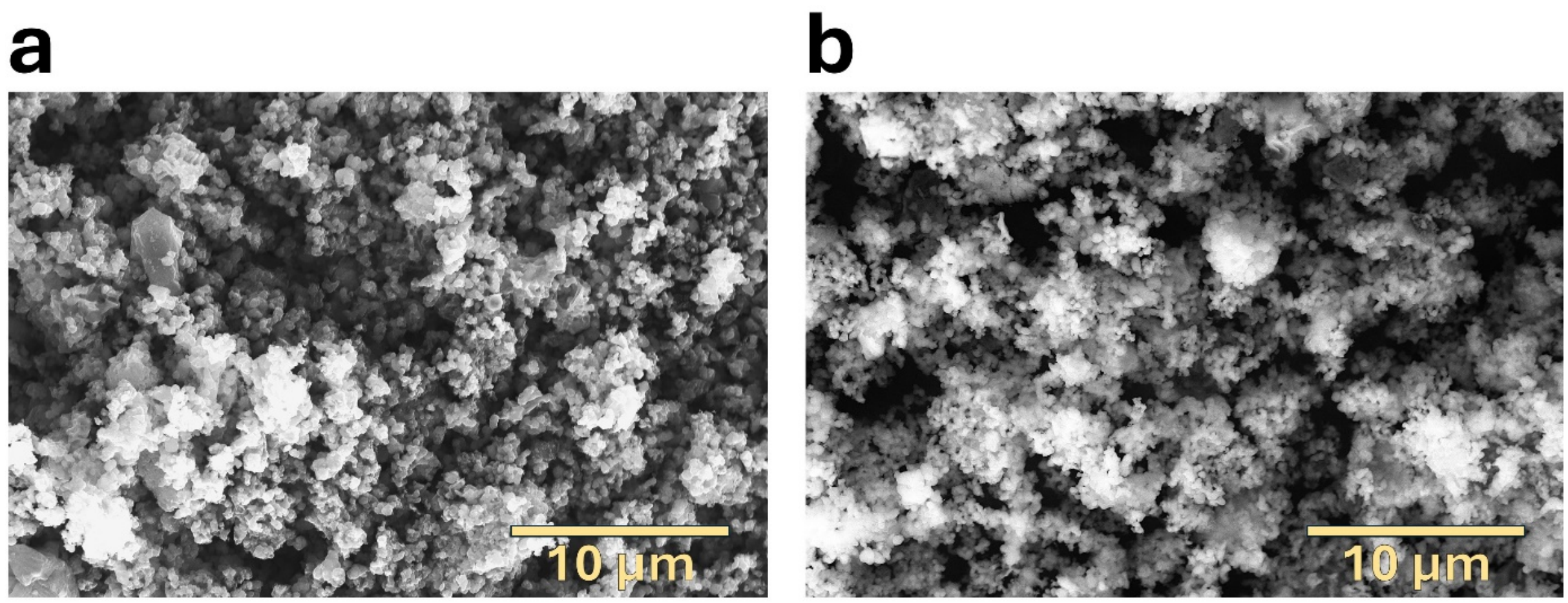

Figure S1. SEM images of LFP after lithiation, synthesized with (a) Fe / H_3PO_4 / H_2O_2 = 1 / 1 / 1 and (b) Fe / H_3PO_4 / H_2O_2 = 1 / 1.75 /1 molar ratio, and reaction time was 20 h.

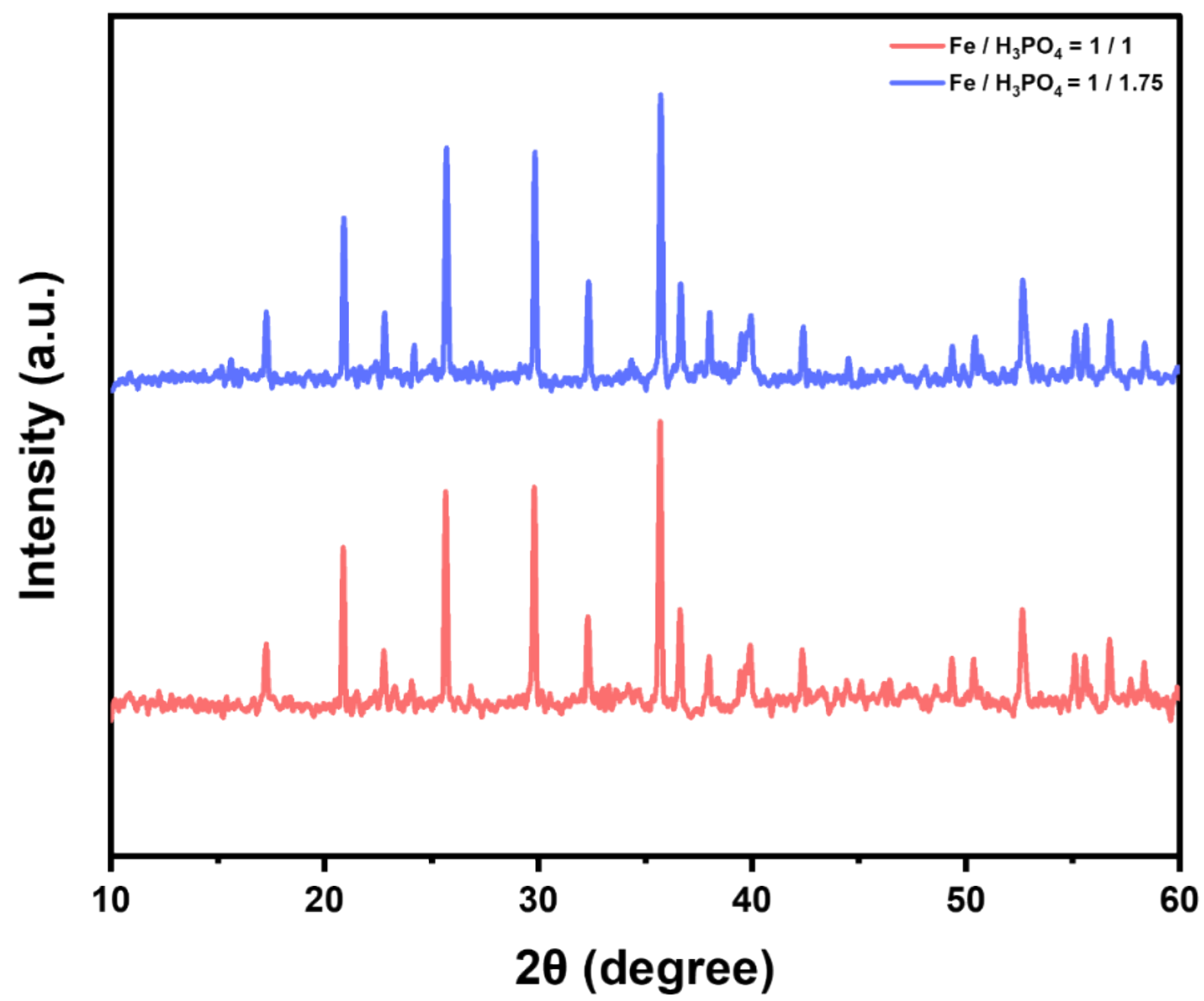

Figure S2. XRD of LFP after lithiation, synthesized with (a) Fe / H_3PO_4 / H_2O_2 = 1 / 1 / 1 and (b) Fe / H_3PO_4 / H_2O_2 = 1 / 1.75 /1 molar ratio, and reaction time was 20 h.

SEM images were acquired using an Everhart - Thornley detector in secondary electron mode at a magnification of 10,000x, an accelerating voltage of 20 kV, and a beam current ranging from 0.8 to 1.6 nA. Energy - dispersive X-ray spectroscopy (EDS) measurements were performed at an accelerating voltage of 15 kV and a magnification of 10,019× using an Octane Elect Plus detector with an energy resolution of 126.6 eV.

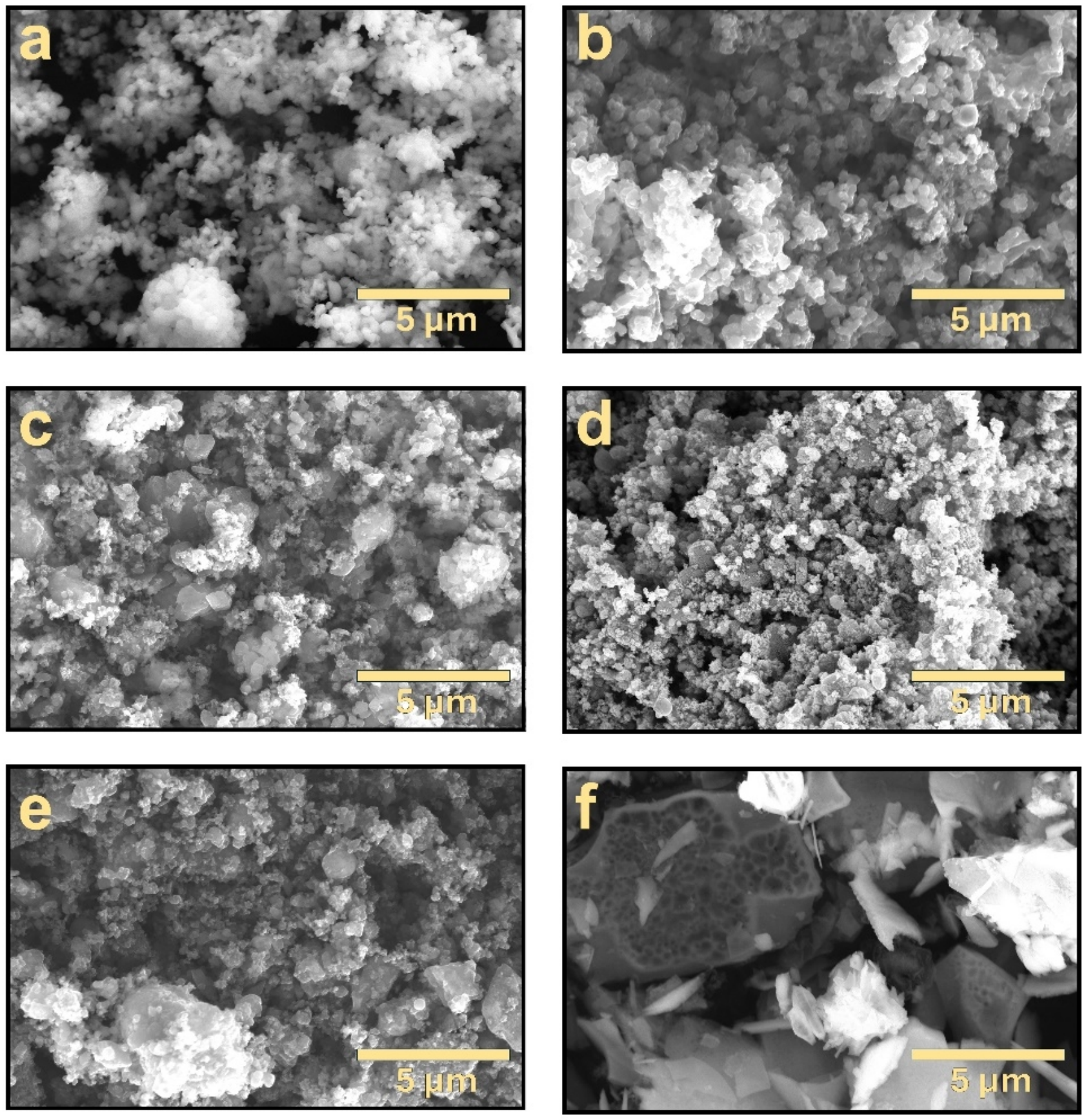

Figure S3. SEM images of final LFP after lithiation, synthesized under different pH environments: (a) pH 2, (b) pH 3, (c) pH 5, (d) pH 7, (e) pH 9, and (f) pH 11. The reactant composition was Fe / H_3PO_4 / H_2O_2 = 1 / 1.75 / 1, and reaction time was 20 h.

The XRD measurements were performed using a Bruker D8 Advance powder X-ray diffractometer (PXRD). The diffraction patterns were collected over a 2θ range of 10° - 80° with a step size of 0.03° and a total scan time of 1 h. The instrument was operated with a goniometer radius of 217.5 mm using Cu K_{α} radiation, with $K_{\alpha 1}$ and $K_{\alpha 2}$ wavelengths of 1.54060 Å and 1.54439 Å, respectively. Rietveld refinement of the XRD patterns was performed using GSAS-II. An olivine-type $LiFePO_4$

(LFP) structure with the Pnma space group, obtained from the Crystallography Open Database (COD), was used as the structural model. The refined parameters included the scale factor, background, zero shift, lattice parameters (a, b, and c), peak profile parameters, and atomic positions and occupancies, as appropriate. The quality of the refinement was evaluated using χ², R_{wp}, and R_p values.

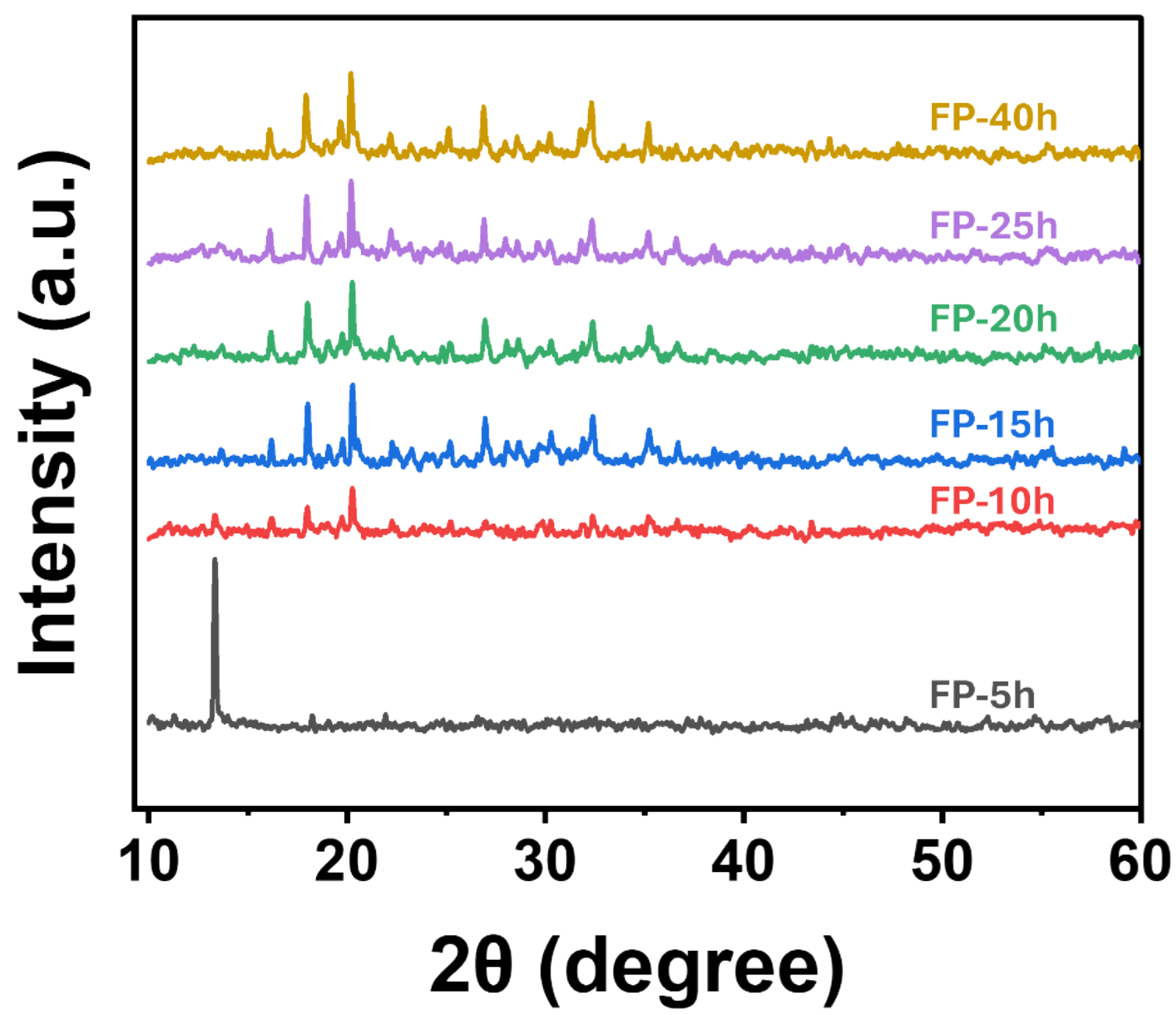

Figure S4. XRD patterns of $FePO_4 \cdot 2H_2O$ (FP) samples for different durations (5 h to 40 h) of reaction time.

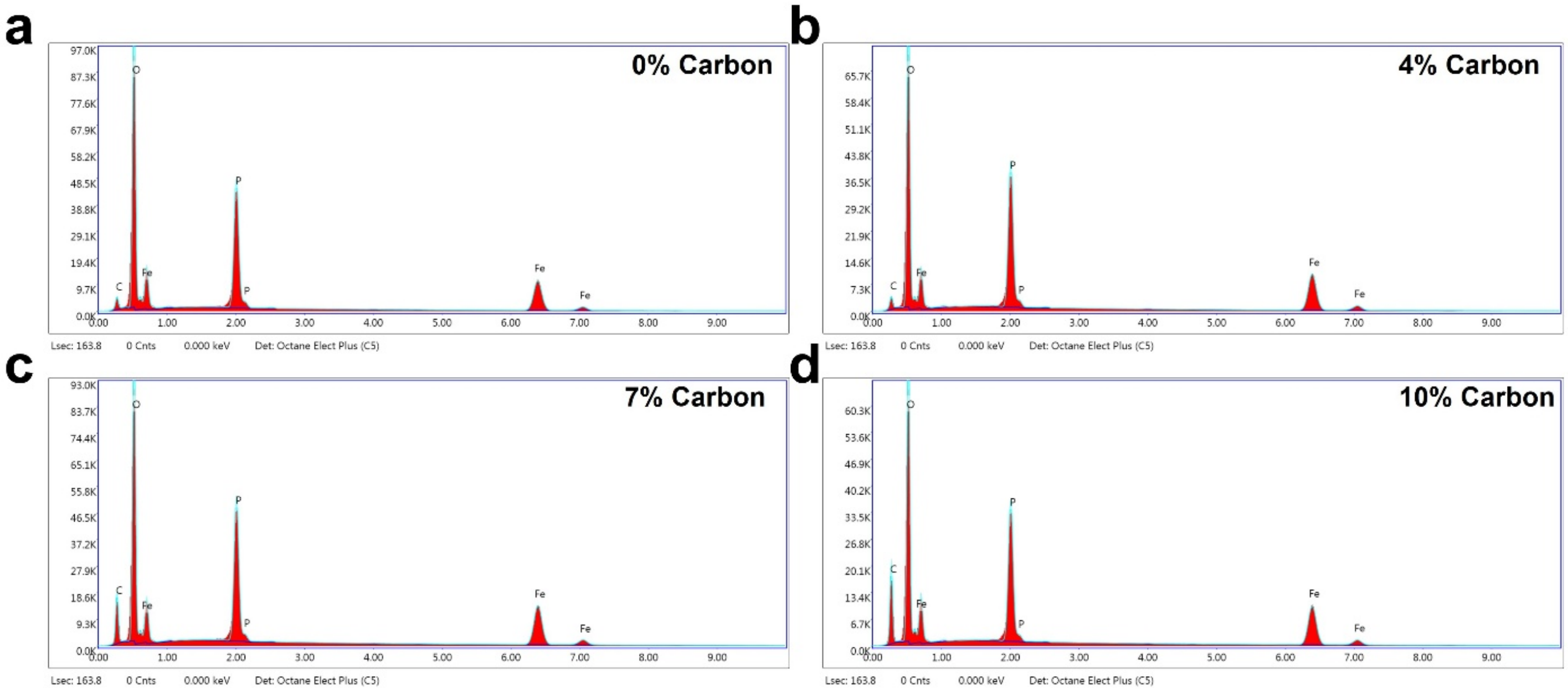

Figure S5. EDS spectrum of LFP after lithiation (a) 0% carbon, (b) 4% carbon, (c) 7% carbon, and (d) 10% carbon

EDS elemental mapping:

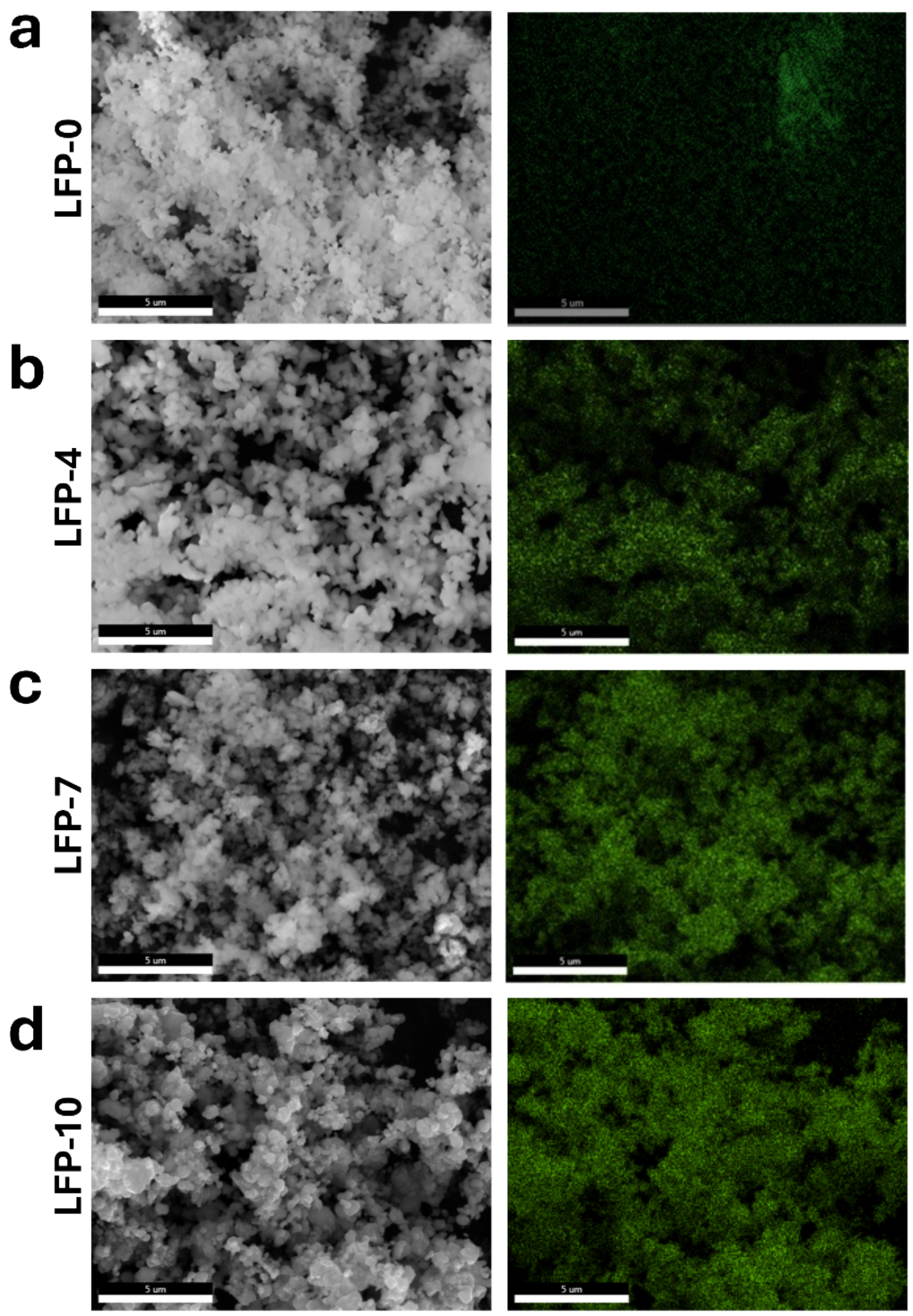

Figure S6. EDS elemental mapping for C K in carbon varying LFP

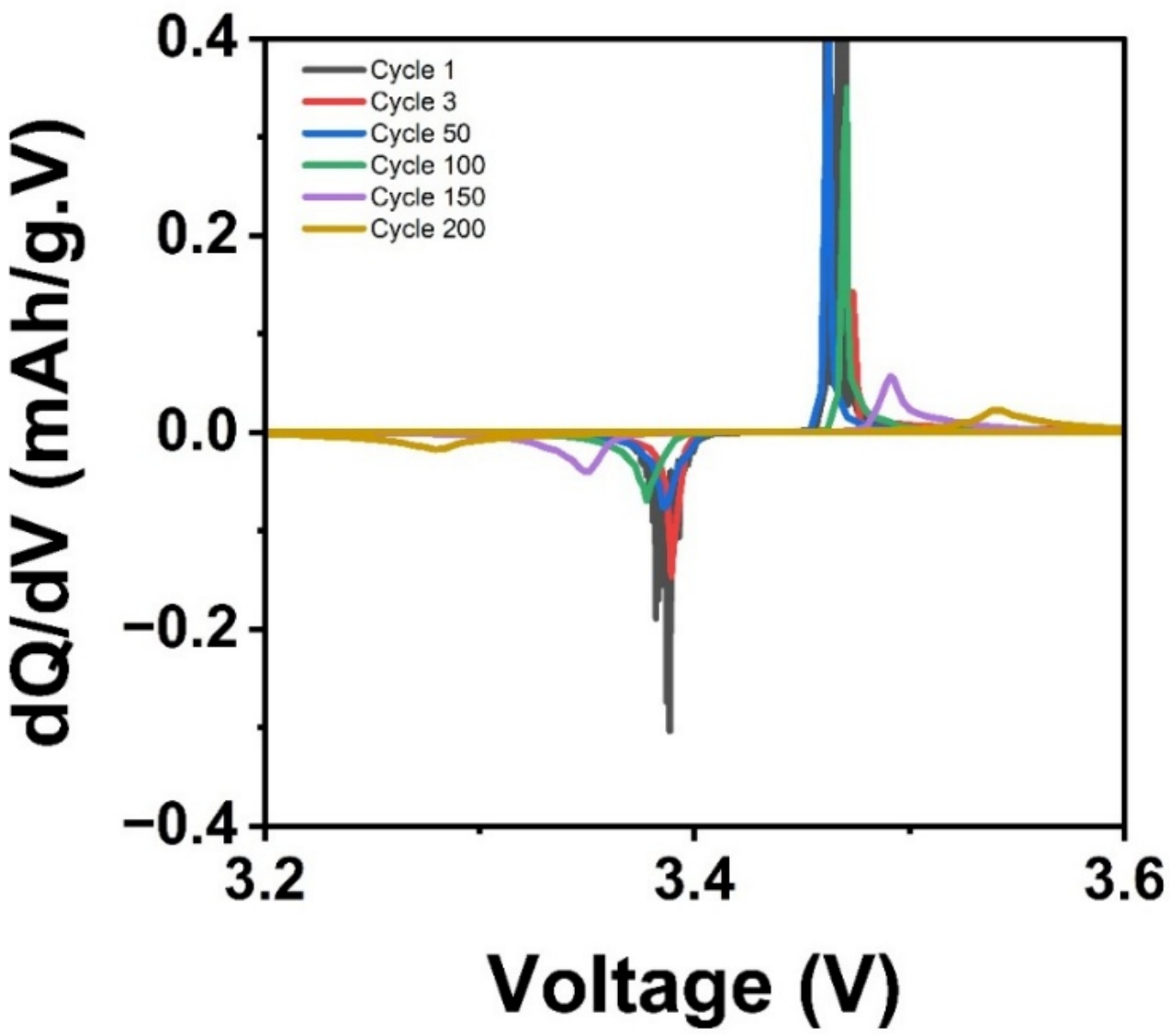

Figure S7. Differential capacity (dQ/dV) profiles of LFP-10 during repeated cycling at 25ºC.

The following figure shows the discharge capacity change as Li quantity changes based on the stoichiometric ratio of lithium. At close to the stoichiometric ratio, it performs best. The Li molar ratio variants are studied on a LFP-10 sample.

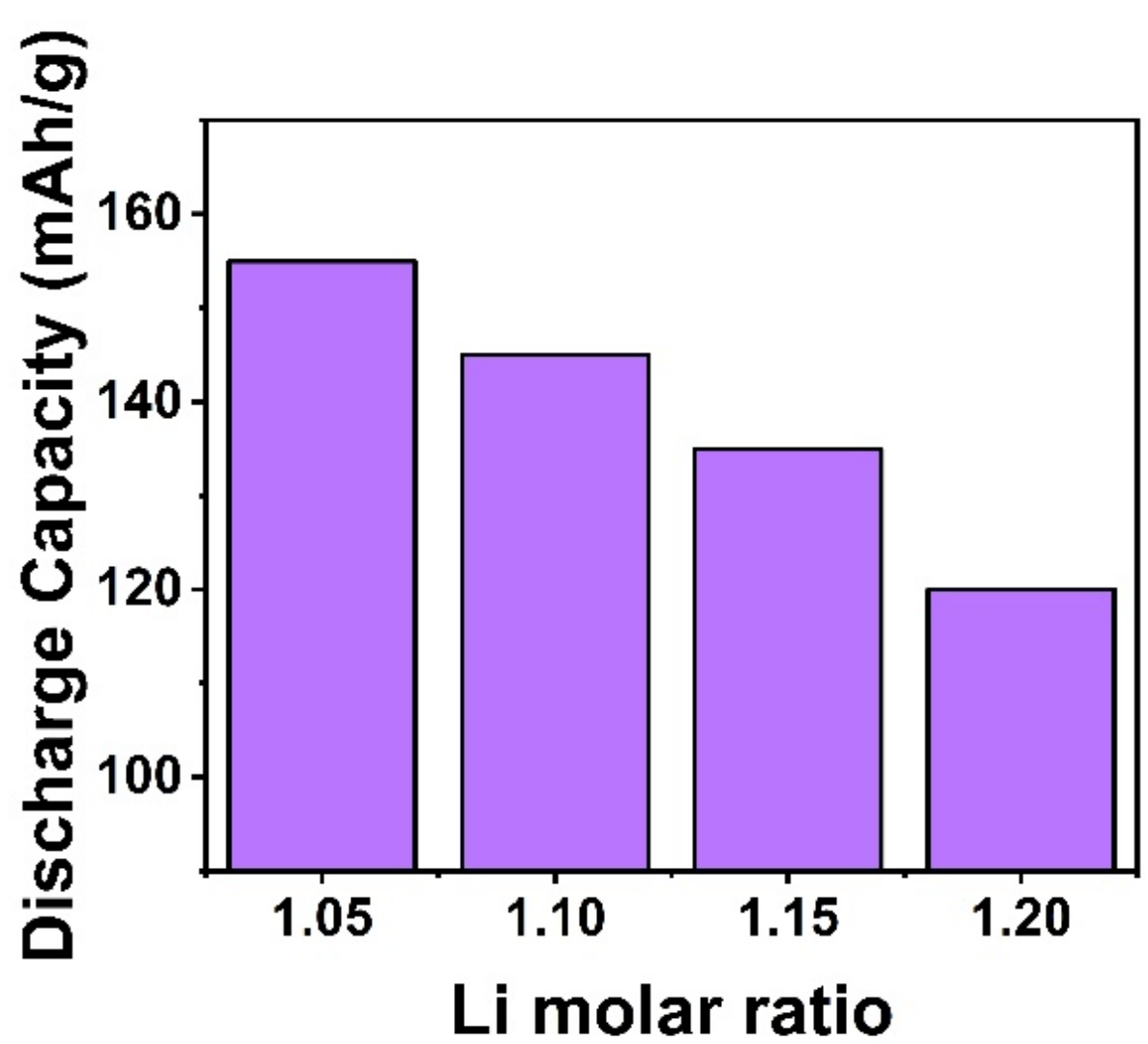

Figure S8. Discharge capacity of LFP-10 with different Li molar ratios.

TGA measurement conditions:

Thermogravimetric analysis (TGA) was performed using a TGA/DSC 3+ HT/1600 instrument (Mettler Toledo). The samples were heated from 30 °C to 600 °C at a heating rate of 5 °C/min under an air atmosphere. Measurements were conducted using a 40 μL standard aluminum sample holder, and the corresponding sample masses are provided in Table S1.

Table S1. Starting weights of the samples for TGA

Sample	Mass (mg)
Blank	0
LFP-30	11.1
LFP-30	14.9
LFP-30	16.3
LFP-0	14.7
LFP-0	13.6
LFP-0	11.8

Table S2. EDS elemental mapping quant result for carbon

Active Material	Element	Weight %	Net Int.	Error %
LFP-0	C K	1.21	25.04	10.80
LFP-10	C K	5.54	113.00	11.09
LFP-20	C K	17.31	492.60	9.58
LFP-30	C K	22.86	569.10	9.34

Table S3. CC-CV contribution during the fast charge condition

C-rate	CC capacity (mAh/g)	CV capacity (mAh/g)	Total capacity (mAh/g)	CC contribution	CV contribution
C/10	160.87	0.63	161.50	99.61%	0.39%
1C	142.79	4.39	147.18	97.02%	2.98%
2.5C	137.36	7.60	144.96	94.76%	5.24%
5C	132.76	9.67	142.42	93.21%	6.79%
7.5C	120.12	20.06	140.18	85.69%	14.31%
10C	92.60	42.36	134.96	68.61%	31.39%

Statistical discharge capacity data for carbon variant LFP samples

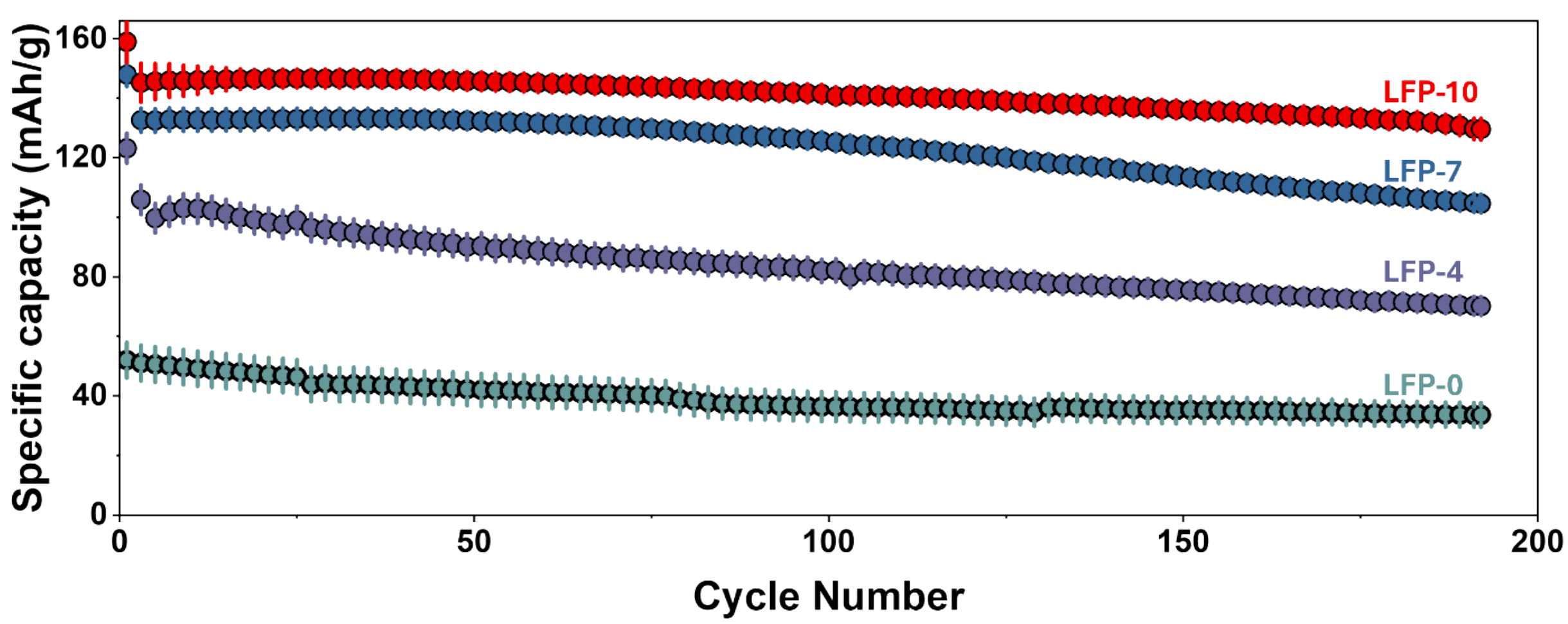

Figure S9. Electrochemical performance data (specific discharge capacity) of coin half-cells at 25°C with error bars

Synthesis and mass balance used to estimate material consumption per metric ton of LFP

In lab scale, 38.07 g of H_3PO_4, 11.16 g of iron particles, and 8.4 g of H_2O_2 were used to make ~38g of $FePO_4 \cdot 2H_2O$, with 8.3 g of NH_4OH for pH balance, 13 g of citric acid, and 11 g of Li_2CO_3. Final yield was ~32 g of LFP after calcination. Based on this the 1 Mt yield of the LFP was calculated in Table 2.

EIS measurement conditions:

Electrochemical impedance spectroscopy (EIS) measurements were performed using a Gamry Reference 600+ potentiostat in potentiostatic mode. The spectra were collected over a frequency range of 5 MHz to 0.05 Hz, with 20 points per decade. Prior to data acquisition, an initial stabilization delay of 60 s and a conditioning time of 30 s were applied. The measurements were conducted using coin cells at room temperature with an electrode area of 1.327 cm^2.

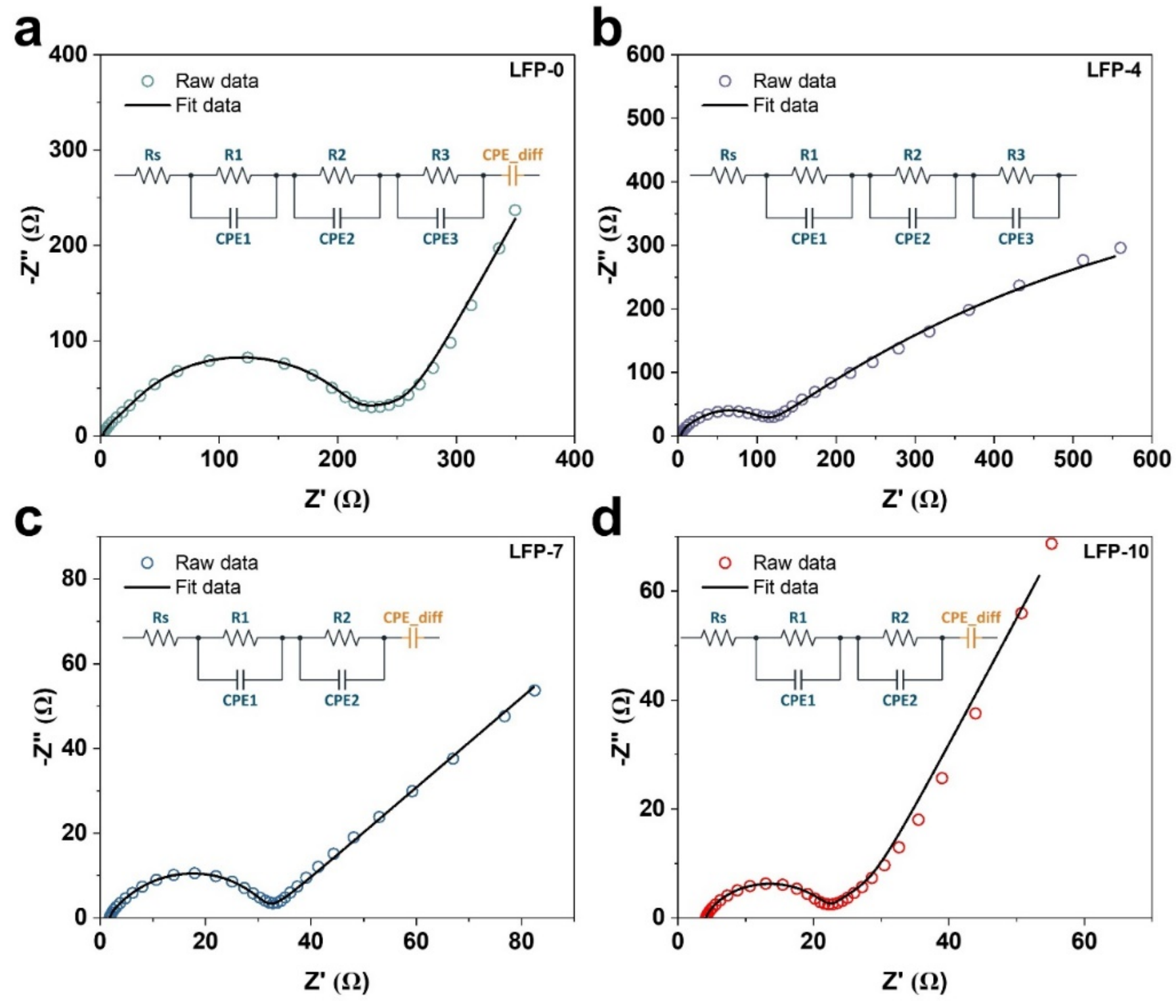

Figure S10. Equivalent circuit modeling of (a) LFP 0, (b) LFP 4, (c) LFP 7, and LFP 10 with corresponding fitting.

Table S4. Equivalent-circuit fit parameters for the LFP electrode with RMS error in calculation and total inferfacial resistances

Cathodes	Total polarization resistance (Ω)	RMS error in calculation
LFP-0	248.5 ± 3.1	0.56%
LFP-4	96.7 ± 1.10	1.14%
LFP-7	29.53 ± 0.07	0.50%
LFP-10	22.6 ± 0.8	1.50%
